# Accuracy of heritability estimations in presence of hidden population stratification

**DOI:** 10.1038/srep26471

**Published:** 2016-05-25

**Authors:** Claire Dandine-Roulland, Céline Bellenguez, Stéphanie Debette, Philippe Amouyel, Emmanuelle Génin, Hervé Perdry

**Affiliations:** 1 CESP, Inserm, Univ. Paris-Sud, Université Paris-Saclay, Villejuif, France; 2 UMR1167 - Labex Distalz, Univ. Lille, Inserm, CHU Lille, Institut Pasteur de Lille, F-59000 Lille, France; 3 Université de Bordeaux, Bordeaux, France; 4 Inserm U1219, Bordeaux, France; 5 CHU de Bordeaux, Bordeaux, France; 6 Inserm, UMR 1078, Brest, France; 7 Université Bretagne Occidentale, Brest, France; 8 Centre Hospitalier Régional Universitaire, Brest, France

## Abstract

The heritability of a trait is the proportion of its variance explained by genetic factors; it has historically been estimated using familial data. However, new methods have appeared for estimating heritabilities using genomewide data from unrelated individuals. A drawback of this strategy is that population stratification can bias the estimates. Indeed, an environmental factor associated with the phenotype may differ among population subgroups. This factor being associated both with the phenotype and the genetic variation in the population would be a confounder. A common solution consists in adjusting on the first Principal Components (PCs) of the genomic data. We study this procedure on simulated data and on 6000 individuals from the Three-City Study. We analyse the geographical coordinates of the birth cities, which are not genetically determined, but the heritability of which should be overestimated due to population stratification. We also analyse various anthropometric traits. The procedure fails to correct the bias in geographical coordinates heritability estimates. The heritability estimates of the anthropometric traits are affected by the inclusion of the first PC, but not by the following PCs, contrarily to geographical coordinates. We recommend to be cautious with heritability estimates obtained from a large population.

The heritability of a quantitative phenotype is the proportion of its variance explained by genetic factors. The concept of heritability can be traced back to the pioneering works of Galton in the nineteenth century^1^; its modern definition is due to Fisher in^2^. Most recent works focus on the narrow-sense heritability which is the proportion of variance explained by additive genetic effects.

Heritability estimates have long been based on family data^3^. Twin studies^4^ were very popular, as they provide an easy way to take into account the shared environment in families, which can bias the estimates if unaccounted for in the analyses. However the possibility of a bias due to difference in shared environment in monozygotic and dizygotic twins remained^4^,^5^.

The research of the genetic polymorphisms causing the variability of the phenotypes with a strong genetic component was carried notably through numerous Genome-Wide Association Studies (GWAS). Hundreds of Single Nucleotide Polymorphisms (SNPs) have been found associated with complex traits^6^,^7^. However the variance explained by these SNPs is lower than the genetic variance predicted by the family studies; the unexplained variance was called the “missing heritability”^8^,^9^,^10^,^11^,^12^,^13^.

The missing heritability problem triggered the development of methods using genome-wide data to obtain heritability estimates from unrelated individuals^13^,^14^. These methods have now mostly superseded family based methods; they are often advocated and perceived as providing unbiased estimates, in particular because, as the individuals are unrelated, there is no shared environment^15^. It was however demonstrated early that the presence of population stratification inflates heritability estimates^16^. A common practice to correct this bias is to include a few Principal Components (PCs) of the estimated kinship matrix in the model with fixed effects^17^,^18^,^19^,^20^,^21^.

The common usage is to include 10 or 20 PCs in the model with fixed effects. There are however no theoretical grounds on which this number of PCs is chosen; more PCs could be included without damaging the estimates. In this paper, we explore the variability of genomic heritability estimates with the number of PCs included as fixed effects in the model, and the ability of this method to effectively correct for population stratification.

We use simulated data to assess the properties of the method, which we also apply on the Three-City (3C) data^22^. The 3C study is a longitudinal study including 9294 French individuals aged 65 years or older, recruited between 1999 and 2001 in the cities of Bordeaux, Dijon and Montpellier. The longitude and latitude of the birth cities provide examples of “purely stratified” traits, which are not determined by the genome but should be very correlated to the first PCs^23^. Thus, the “naive” genomic heritability estimates of these traits should be artificially high; including PCs in the model should reduce these estimates. Our aim is to find how many PCs have to be included to get a genomic heritability estimate close to zero.

We also analyse anthropometric phenotypes: height which is the historical example of a highly heritable trait^2^,^24^, weight, Body Mass Index (BMI), head circumference and waist-to-hip ratio which are also expected to display significant heritability. GWAS studies discovered many variants associated with human height^25^,^26^,^27^, and with obesity related traits such as weight, BMI, waist or hip circumference and waist-to-hip ratio^28^,^29^,^30^,^31^,^32^,^33^. The heritability of all these traits have been estimated multiple times through twin studies, familial studies, or genomic data; Table 1 summarizes some estimates from the literature. We compute their genomic heritability on the 3C data set, and consider how it evolves when including PCs with fixed effects.

## Results

Using linear mixed models, we estimate variance components and heritabilities *h*^2^ of several traits. The decompositions of phenotypic variance are presented in graphics with number of Principal Components included with fixed effects in the model as abscissae and the proportion of explained variance as ordinates. The estimated proportion of variance explained by the fixed effects is displayed in dark gray. The light gray and white colors are respectively the estimated proportions of genetic and residual variances.

For the 3C phenotypes, we also give Tables with numerical value of the parameter estimates, their standard errors, and the likelihood ratio test for *H*_0_: *h*^2^ = 0.

### Simulated data

Phenotypes were simulated under the linear mixed model, with a non-zero effect on the first 10 PCs (see details in Methods section). They were analysed with a number *p* of PCs included in the model with fixed effects varying from 0 to 2000.

**Figure 1:**
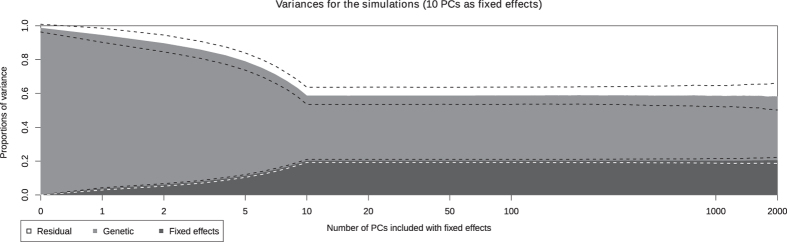
Estimated proportions of variance for the simulated data, depending on the number of PCs included in the model (log-scale). The white, light gray and dark gray are respectively the residual, genetic and fixed effects estimated means on 100 replicates. The dashed lines represent the mean ±1 standard deviation.

Figure 1 displays the mean of estimated variance proportions, and their standard deviations, computed on 100 simulation replicates. From 0 to 2000 PCs are included with fixed effects. We note that as soon as 10 PCs or more are included in the model, the true proportions of variance (20% for the PCs, 40% for each of the random terms) are well estimated (with a standard error close to 0.05). In contrast, when the number of included PCs is lower than 10, the proportion of genetic variance is overestimated. When up to 2000 PCs are included, the means of estimates are stable, while the standard deviation increase slowly.

### Three-City data

Variance estimations have also been made on several quantitative traits from the 3C study. First of all, we considered as quantitative phenotypes the longitude and the latitude of the birth cities. Geographical coordinates of all birth cities are displayed on the map of France in the first panel of Supplementary Fig. S1. We also estimated the heritability of several anthropometric phenotypes: height, weight, BMI, waist-to-hip ratio and head circumference.

The mean and the standard deviation of each analyzed variable are given in Table 2, stratified on the sex. The sex has been included as a covariate when estimating the heritability of the anthropometric phenotypes.

#### Longitude and latitude

**Figure 2:**
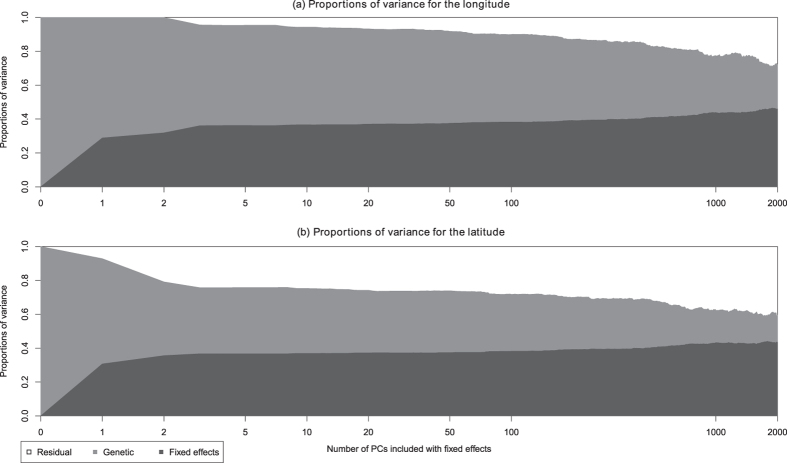
Estimated proportion of variance for the geographical coordinates, depending on the number of PCs included in the model (log-scale). The white, light gray and dark gray are respectively the residual, genetic and fixed effects variances.

The variance decompositions of longitude and latitude are displayed in the two panels of Fig. 2, where we included up to *p* = 2000 PCs in the model with fixed effects. When no PCs are included in the model, it is estimated that 100% of the variance is genetic both for longitude and latitude. When adding PCs to the model, this estimated proportion decreases slowly for the longitude (first panel of Fig. 2). It is estimated close to 54% for *p* = 50 PCs, and to 26% for *p* = 2000. In contrast, the estimated proportion of genetic variance of latitude decreases sharply (to 39% of the variance) with the first few PCs included in the model; it then decreases slowly, as observed for latitude: it is still close to 36% for *p* = 50 PCs, decreasing to 16% for *p* = 2000. For both geographical coordinates, the first PC explains a non-negligible proportion of variance (29% and 31% for longitude and latitude respectively). Once the first PC is included, the proportion of variance explained by the PCs increases slowly with *p*. This observation is consistent with the correlation values of the geographical coordinates with the first few PCs (Supplementary Fig. S2).

Precise figures are given in Tables 3 and 4 for longitude and latitude heritability respectively. These Tables give, for various values of *p*, the estimates of *σ*^2^, *τ*, and *h*^2^, together with their standard error, and the likelihood ratio test for the null hypothesis *H*_0_: *h*^2^ = 0. We first note that for all values of *p* up to *p* = 2000, the Likelihood Ratio Test (LRT) is significant for the heritability of both longitude and latitude (note that the LRT asymptotically follows a 
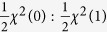
 mixture^34^; the 5% significance threshold is 2.70).

We also note that the standard error of the various estimates increases with *p*, but within reasonable bounds: for example the estimated heritability of the longitude is 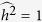 with standard error 0.04 for *p* = 0, 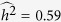 with standard error 0.09 for *p* = 1000, and 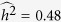 with standard error 0.12 for *p* = 2000.

#### Anthropometric traits

We analysed height, weight, BMI, head circumference and waist-to-hip ratio. For these traits, sex and age were included in the model as covariates.

**Figure 3:**
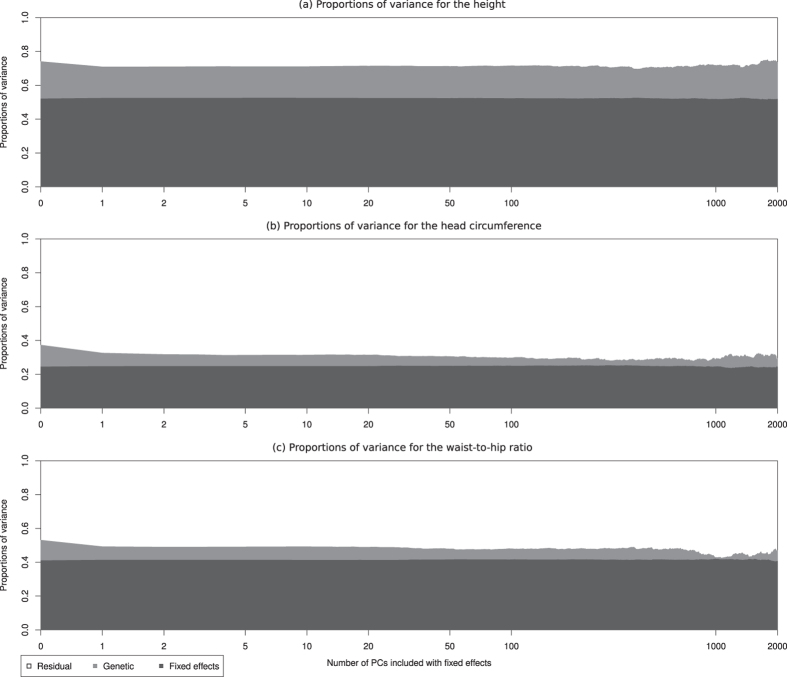
Estimated proportion of variance for (**a**) height, (**b**) head circumference, and (**c**) waist-to-hip-ratio, depending on the number of PCs included in the model (log-scale). The white, light gray and dark gray are respectively the residual, genetic and fixed effects variances.

In Fig. 3, the estimated proportions of variance for height, head circumference and waist-to-hip ratio are displayed. Sex and age alone are responsible for 52%, 24% and 41% of the variance of height, head circumference and waist-to-hip ratio respectively. Without population stratification correction (*p* = 0), the genetic variance is 22%, 13% and 12% of the total variance; the inclusion of a single PC in the model (*p* = 1) decreases these values to 19%, 8% and 8% respectively. Including up to *p* = 50 PCs does not modify much these proportions. For *p* up to 2000, the variance proportion estimates fluctuate around the previously cited values.

The estimated proportions of variance for weight and BMI are displayed in Supplementary Fig. S3: the inclusion of PCs with fixed effect does not impact much the estimated proportions of genetic variance, which are 16% and 19% respectively.

Table 5 gives for the five anthropometric phenotypes and various values of *p*, the estimates of *σ*^2^, *τ*, and *h*^2^, together with their standard error, and the likelihood ratio test for the null hypothesis *H*_0_: *h*^2^ = 0.

Without stratification correction, height heritability is estimated to 46% with standard error of 6.1%; as soon as one PC is included in the model, this value drops to 39% (standard error 6.6%). Inclusion of more PCs (up to *p* = 20) almost does not change the estimated values nor their standard errors. The likelihood ratio tests for *h*^2^ = 0 are significant.

Heritability of weight and BMI seem to be almost unaffected by population stratification correction. In the two cases, heritability is significantly positive. It is estimated close to 22% (standard error 6%) and 19% (standard error 6%).

Head circumference heritability is estimated to 17% (standard error 5.7%) without population stratification correction. With inclusion of *p* = 1 PC in the model, it is only 10%, which drops to 9% for *p* = 2 (with a standard error of 6.3% or 6.4%). The likelihood ratio test is no longer significant.

Waist-to-hip ratio heritability is estimated to 20% (standard error 6.1% without population stratification correction. This value drops to 13% (standard error 6.7%) with the inclusion of one PC in the model. The likelihood ratio tests stay significant.

## Discussion

Our analyses of simulated data with up to 2000 PCs included as fixed effect show that the heritability estimates precision are not much impacted when an important number of PCs are included in the model with fixed effects. Of course, the size of the sample matters, however as soon as a few thousands individuals are included in the analysis, taking *p* = 100 or 500 is not harmful for the precision of the estimates. There is no practical reason to limit to small values of *p*.

We were surprised to obtain a genomic heritability estimate of 100% for latitude and longitude: as they are correlated to the first few PCs, we were expecting a positive heritability estimate, but not that large. We were also expecting the estimated heritability to vanish (or at least to become very small) after inclusion of a few PCs in the model. Instead of that, if the latitude heritability drops to 68% after inclusion of the first two PCs, adding more PCs only results in a slow decrease, and the heritability remains highly significant. The longitude behaves in a worse manner, as there is no initial drop as observed for the longitude. In both cases, the slow decrease of heritability is accompanied by a slow increase of the proportion of total variance due to the PCs included with fixed effects. The Likelihood Ratio Test (LRT) shows that the heritability is significantly positive for all values of *p*.

Browning *et al*.^16^ obtained similar results with simulated case/control data based on the WTCCC case/control data, with an extremely disequilibrated ascertainment scheme in which 90% of individuals from Scotland and Wales were assigned to be cases, and only 10% of individuals from England, the controls being the remaining individuals. In their response, Goddard *et al*.^15^ pointed out that this was an extreme scenario; it is however realistic to imagine that a quantitative trait is under the influence of an environmental factor which varies with latitude or longitude. The heritability estimate of such a trait would be severely overestimated.

Heritability estimates obtained for anthropometric traits are globally compatible with the results from the literature. It is interesting to note that height and waist-to-hip ratio seem sensitive to population stratification, even if marginally. Head circumference is more affected, as its estimated heritability drops from 17% to 9% with the inclusion of the first two PCs, and the LRT is no longer significant. All these traits are weakly but significantly correlated with the geographical coordinates of the birth cities (cf Supplementary Table S5), but so is BMI which does not display this behaviour. We note that when including more PCs in the analysis of these traits, there is no linear trend comparable to the one observed in the case of the geographical coordinates. This can bring back some confidence in the method, which was dented by the previous analyses.

We performed additional analyses were we included the geographical coordinates as covariates when analysing height, head circumference, and waist-to-hip ratio (Supplementary Fig. S4 and Supplementary Table S1): the drop in heritability when the first PCs are added is no longer observed, which seems to indicate that the effect of the first PCs was due to a geographical stratification. Heritability of height drops to 37% (instead of 39%), which is a marginal change; as previously, heritability of head circumference is not significantly positive. Heritability of waist-to-hip ratio is estimated close to 8%, and is no longer significantly positive, while it was previously estimated to 13%, significant with *p* = 2: this falls in line with our previous findings on the fact that the inclusion of the first PCs may not be sufficient to fully correct for population stratification. One should consider seriously to include the geographical coordinates of the place of birth with fixed effect in the model, when they are available.

Epidemiologists use to take into account possible center effects by including indicator variables for the centers. We performed an additional analysis of height, head circumference and waist-to-hip ratio with fixed effects for the centers (Supplementary Fig. S5 and Supplementary Table S2). As observed for the previous analysis incorporating geographical coordinates, the drop in heritability after including the first PCs almost disappears (although not completely). The final value after inclusion of 10 PCs is however somewhat higher than the one obtained with the geographical coordinates. We also performed an analysis on the individuals from the largest center, Dijon, alone (Supplementary Fig. S6 and Supplementary Table S3). The drop in heritability after including the first PC is then quite noticeable. This discards the hypothesis that the impact of the PCs on the heritability estimates is due to a center effect. Note however that it is impossible to tell whether the first PCs effect is due to some geographic environment, or to some genes with a north-south or west-east allelic gradient.

We made the choice of using LD-pruned data to compute the PCs included for population stratification. We could have used the whole data to this aim; this does not alter significantly our conclusions. The results of such analyses are displayed in the Supplementary Information 2.

A procedure has been proposed to detect the presence of cryptic relatedness and population stratification^35^. It consists in computing, in one hand, the 22 heritabilities due to each chromosome, one at time, and on the other hand, the same quantities by analysing all the chromosomes together. In absence of cryptic relatedness and of population stratification, these two estimates should be (roughly) the same. The procedure then regresses the difference between those two estimates on the length of the chromosomes. A significantly positive intercept is interpreted as due to the presence of cryptic relatedness, while a significantly positive slope is interpreted as due to population stratification. We applied this procedure on all real data (Supplementary Table S4). This procedure diagnoses well the presence of a population stratification for longitude, latitude, height, head circumference, and waist-to-hip ratio. However, for anthropometric traits, when one PC is added in the model, the procedure would conclude that the population stratification is correctly taken into account. Also, when analysing the latitude, after the inclusion of 10 PCs, the slope is only borderline significant. This suggests that heritability estimates of a trait depending on the latitude, for example through the sun exposure, could have an important bias which would be difficult to detect by this method.

A recent work argued that considering the genotype matrix as fixed in the linear mixed model, while in reality the genotypes are sampled from the population, is a cause of unreliability of genomic heritability estimates, and that this is aggravated by the presence of population stratification^36^; the conclusions of this work are debatable^37^. However, and more generally, the assumptions of the linear mixed model are unrealistic^38^. They certainly are not fulfilled when it comes to the geographical coordinates of the birth cities; we do not know to what extent they are more realistic for other traits. This alone should compel us to take any genomic heritability estimate with a grain of salt.

## Methods

### Estimation of the heritability

We consider the linear mixed model


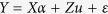


with 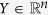 a vector of phenotypes, 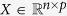 the matrix of covariates included with fixed effects, 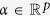 the fixed effect vector, 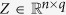 the standardized genotype matrix, 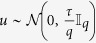 the genetic effect vector 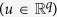 and 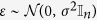 the error vector.

The variance components *τ* and *σ*^2^ are estimated by Restricted Maximum Likelihood (REML)^39^,^40^,^41^,^42^. We used the R package Gaston^43^ and GCTA^44^. Example of commands are given in Supplementary Methods (Paragraph 1).

The heritability is usually estimated by 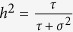 – which ignores the variance of the phenotype which is explained by the covariates included in *X*. This is tantamount to defining the heritability as the proportion of genetic variance in the phenotype variance yet unexplained by known covariates, as in ref. 9. However, it is also possible to split the variance of *Y* in three parts: the variance explained by the covariates in *X*, the genetic variance *τ*, and the remaining variance *σ*^2^.

Estimating the variance due to the covariates by the empirical variance of the components of 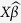 would result in upward biased estimates. We show in Supplementary Methods (Paragraph 2) how to estimate it without bias. We will report both *h*^2^ and this decomposition of the variance in three components.

### Correcting for population stratification

To correct for population stratification, we include the first *p* principal components of the kinship matrix estimated only on a submatrix *Z*_1_ of *Z*, obtained by pruning SNPs from *Z*, to retain approximately 50000 SNPs in low linkage disequilibrium^45^.


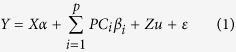


with *PC*_*i*_ the *i*^th^ principal component vector (*n* × 1) and *β*_*i*_ the fixed effect of the *i*^th^ PC.

We will use values of *p* varying from 0 to 2000 (for *n* = 5793).

### The Three-City genomic data

The 3C study^22^ is an ongoing French population-based longitudinal study which started in 1999. Participants were randomly selected from electoral rolls of the cities of Bordeaux, Dijon and Montpellier. To be eligible, they had to be aged 65 years or older, living in one of the recruitment cities, and not institutionalized. A total of 9294 individuals are included. Participants were genotyped with Illumina Human610-Quad BeadChip in the Centre National de Génotypage as described in ref. 46. The 3C data can be shared for an ancillary study, subject to approval by the 3C-Study Steering Committee (http://www.three-city-study.com/genetic-studies.php).

Quality Control was performed using PLINK^47^ as described hereafter. Duplicate individuals and individuals with a discordance between genetic and clinical sex were discarded. In the sequel, only autosomal SNPs were considered. Individuals with more than 5% of missing genotypes, or with a proportion of heterozygous genotypes more than 3 standard deviations away from the observed mean were removed. Individuals with non-European ancestry were excluded using Principal Component Analysis on Hapmap individuals. Individuals known to be born outside mainland France were removed. To eliminate cryptic relatedness, we removed an individual from all pairs with a genetic relatedness superior to 0.025. Finally, SNPs with a call-rate below 99% or Hardy-Weinberg threshold of 10^−8^ were removed.

After Quality Control, there are 5793 individuals (1499, 3676 and 618 from Bordeaux, Dijon and Montpellier respectively) and 509931 autosomal SNPs left.

The Principal Components for population stratification correction are computed on LD-pruned data, where we kept only SNPs with a minor allele frequency higher than 5%, and in mutual LD inferior to 0.1. We also removed SNPs in the long-range LD regions defined in ref. 48. The final LD-pruned dataset includes 49277 SNPs. The distribution of the first two PC depending of recruitment cities are given in Supplementary Fig. S1; the first PC correlates with the collection center. This observation is not surprising, as the geographical coordinates differ from one center to another.

Two types of traits are available; the longitude and the latitude of the birth cities which were retrieved from their zipcode (see first panel of Supplementary Fig. S1) and several anthropometric phenotypes: height, weight, BMI, waist-to-hip ratio and head circumference.

### Simulated phenotypes

We simulated phenotypes according to the model (1), using the 3C genomic data: *Z* is the 5793 × 509931 matrix of standardized genotypes, and the PCs are computed on the LD-pruned data. The *p* = 10 first principal components are included. The coefficients *β*_1_, …, *β*_*p*_ have been chosen such that each PC explains 2% of the phenotype variance, (thus, the 10 PCs together explain 20% of the variance). The remaining variance is equally distributed between genetic and environmental effects (thus, *σ*^2^ = *τ, h*^2^ = 0.5, and the proportion of total variance is 40% for each).

## Additional Information

**How to cite this article**: Dandine-Roulland, C. *et al*. Accuracy of heritability estimations in presence of hidden population stratification. *Sci. Rep.* **6**, 26471; doi: 10.1038/srep26471 (2016).

## Supplementary Material

Supplementary Information

## Figures and Tables

**Table 1:** Heritability estimations in the litterature.

	**Family data**	**Twin data**	**Genomic data**
Height	0.92^49^	0.68 to 0.94^50^,^51^,^52^,^53^	0.44 to 0.62^14^,^35^,^53^,^54^,^55^,^56^,^57^
Weight	–	0.37^53^	0.19^35^, 0.26^53^
BMI	0.24 to 0.81 (mean 0.46)^58^	0.47 to 0.90 (mean 0.75)^58^	0.16 to 0.27^35^,^53^,^55^,^57^
		0.28^53^	
		0.45 to 0.84^59^	
Waist circumference	–	0.15^53^	0.16^53^, 0.17 (men or women)^57^,†
Hip circumference	–	–	0.23 (men)†, 0.19 (women)^57^,†
Waist-to-hip ratio	–	–	0.16 (men)†, 0.18 (women)^57^,†
Head circumference	0.66^60^	0.75^59^	–
Skeletal traits(including head circumference)	–	0.59^52^	–

^†^Adjusted on the BMI and stratified on the sex. Ref. 58 is a meta-analysis of 88 twin studies and 27 family studies.

**Table 2:** Descriptive statistics of the 3C quantitative traits.

**Phenotype**	**Men (*****N***** = 2298)**			**Women (*****N***** = 3495)**			**All (*****N***** = 5793)**		
	**Mean**	**Sd**	***n***	**Mean**	**Sd**	***n***	**Mean**	**Sd**	***n***
Age	74.15	5.56	2298	74.39	5.49	3495	74.30	5.52	5793
Latitude	46.78	1.73	2090	46.76	1.63	3171	46.77	1.67	5261
Longitude	3.32	2.40	2090	3.35	2.45	3171	3.34	2.43	5261
Height	169.58	6.35	2290	156.60	6.17	3461	161.77	8.91	5751
Weight	75.58	11.27	2292	62.58	11.32	3485	67.74	12.97	5777
BMI	26.27	3.53	2288	25.52	4.36	3457	25.82	4.06	5745
Head circumference	57.75	2.05	2243	55.37	2.07	3414	56.32	2.37	5657
Waist-to-hip ratio	0.95	0.07	2117	0.84	0.07	3180	0.88	0.09	5297

**Table 3:** Model parameter estimates for the longitude and their standard error, depending on the number of PCs included in the model, likelihood ratio test statistics (LRT) to test significance of heritability and their *p*-values^34^.

	**LRT**	***p*****-value**	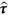 **(se)**	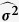 **(se)**	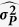 **(se)**	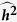 **(se)**
0 PC	1892.63	<1e-40	4.34 (0.085)	0.00029 (0.089)	4.34 (0.084)	1.000
1 PC	563.65	<1e-40	3.83 (0.075)	0.00025 (0.069)	3.83 (0.075)	1.000
2 PCs	405.08	<1e-40	3.71 (0.072)	0.00025 (0.065)	3.71 (0.072)	1.000
3 PCs	221.81	<1e-40	3.28 (0.069)	0.24 (0.061)	3.53 (0.069)	0.931 (0.060)
4 PCs	219.54	<1e-40	3.27 (0.069)	0.26 (0.061)	3.52 (0.069)	0.928 (0.060)
5 PCs	219.56	<1e-40	3.27 (0.069)	0.25 (0.061)	3.52 (0.069)	0.928 (0.060)
10 PCs	204.70	<1e-40	3.19 (0.069)	0.32 (0.061)	3.50 (0.068)	0.910 (0.061)
20 PCs	193.57	<1e-40	3.12 (0.069)	0.37 (0.061)	3.49 (0.068)	0.894 (0.062)
50 PCs	177.83	<1e-40	3.00 (0.068)	0.46 (0.061)	3.46 (0.068)	0.868 (0.063)
100 PCs	157.09	2.4e-36	2.87 (0.068)	0.56 (0.061)	3.43 (0.067)	0.838 (0.065)
500 PCs	87.59	4.0e-21	2.35 (0.070)	0.97 (0.063)	3.32 (0.068)	0.707 (0.073)
1000 PCs	43.24	2.4e-11	1.90 (0.072)	1.30 (0.065)	3.20 (0.069)	0.594 (0.087)
2000 PCs	14.00	9.1e-5	1.51 (0.083)	1.61 (0.072)	3.12 (0.077)	0.485 (0.124)

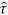
 is the estimated genetic variance, 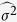 the estimated residual variance, 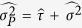 the estimated total variance, and 
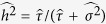
 estimated heritability.

**Table 4:** Model parameter estimates for the latitude and their standard error, depending on the number of PCs included in the model, likelihood ratio test statistics (LRT) to test significance of heritability and their *p*-values.

	**LRT**	***p*****-value**	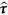 **(se)**	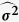 **(se)**	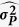 **(se)**	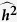 **(se)**
0 PC	1854.15	<1e-40	2.05 (0.040)	0.00014 (0.039)	2.05 (0.040)	1.000
1 PC	295.37	<1e-40	1.60 (0.035)	0.18 (0.031)	1.78 (0.035)	0.897 (0.054)
2 PCs	126.24	1.4e-29	1.14 (0.033)	0.55 (0.031)	1.69 (0.033)	0.676 (0.061)
3 PCs	98.87	1.3e-23	1.03 (0.033)	0.64 (0.031)	1.66 (0.032)	0.616 (0.063)
4 PCs	99.13	1.2e-23	1.03 (0.033)	0.64 (0.031)	1.67 (0.032)	0.617 (0.063)
5 PCs	99.37	1.0e-23	1.03 (0.033)	0.64 (0.031)	1.67 (0.032)	0.618 (0.063)
10 PCs	95.50	7.4e-23	1.00 (0.033)	0.65 (0.031)	1.66 (0.032)	0.608 (0.063)
20 PCs	88.35	2.7e-21	0.98 (0.033)	0.68 (0.031)	1.66 (0.032)	0.589 (0.063)
50 PCs	83.73	2.8e-20	0.96 (0.033)	0.69 (0.031)	1.65 (0.032)	0.582 (0.064)
100 PCs	68.81	5.4e-17	0.89 (0.033)	0.75 (0.031)	1.64 (0.032)	0.543 (0.066)
500 PCs	38.96	2.2e-10	0.73 (0.034)	0.86 (0.031)	1.59 (0.033)	0.459 (0.074)
1000 PCs	15.26	4.7e-5	0.52 (0.035)	1.02 (0.032)	1.54 (0.033)	0.338 (0.086)
2000 PCs	5.22	0.0112	0.44 (0.042)	1.11 (0.037)	1.55 (0.038)	0.284 (0.122)

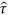
 is the estimated genetic variance, 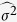 the estimated residual variance, 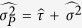 the estimated total variance, and 
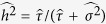
 estimated heritability.

**Table 5:** Model parameter estimates for the anthropometric phenotypes and their standard error, depending on the number of PCs included in the model, likelihood ratio test statistics (LRT) to test significance of heritability and their *p*-values.

**Phenotype**		**LRT**	***p*****-value**	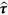 **(se)**	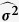 **(se)**	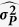 **(se)**	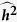 **(se)**
Height	0 PC	68.80	5.6e-17	17.48 (0.719)	20.64 (0.689)	38.12 (0.687)	0.459 (0.061)
	1 PC	36.32	8.4e-10	14.70 (0.712)	23.12 (0.690)	37.83 (0.685)	0.389 (0.066)
	5 PCs	36.57	7.4e-10	14.73 (0.712)	23.03 (0.690)	37.76 (0.685)	0.390 (0.066)
	10 PCs	36.60	7.2e-10	14.77 (0.712)	23.00 (0.690)	37.78 (0.686)	0.391 (0.066)
	20 PCs	37.87	3.8e-10	15.10 (0.715)	22.75 (0.691)	37.85 (0.686)	0.399 (0.066)
Weight	0 PC	13.92	9.6e-5	28.24 (2.32)	97.23 (2.32)	125.47 (2.11)	0.225 (0.062)
	1 PC	13.34	1.3e-4	27.91 (2.32)	97.53 (2.32)	125.44 (2.12)	0.222 (0.062)
	5 PCs	12.50	2.0e-4	27.19 (2.32	98.21 (2.32)	125.40 (2.13)	0.217 (0.062)
	10 PCs	12.07	2.6e-4	26.77 (2.32)	98.59 (2.32)	125.36 (2.13)	0.214 (0.062)
	20 PCs	12.10	2.5e-4	26.91 (2.32)	98.56 (2.32)	125.47 (2.13)	0.214 (0.063)
BMI	0 PC	10.44	6.2e-4	3.23 (0.302)	13.09 (0.302)	16.31 (0.304)	0.198 (0.063)
	1 PC	9.26	1.2e-3	3.11 (0.302)	13.19 (0.302)	16.30 (0.304)	0.191 (0.064)
	5 PCs	8.56	1.7e-3	3.00 (0.301)	13.29 (0.303)	16.30 (0.304)	0.184 (0.064)
	10 PCs	8.47	1.8e-3	2.99 (0.302)	13.30 (0.303)	16.30 (0.304)	0.184 (0.064)
	20 PCs	8.52	1.8e-3	3.01 (0.302)	13.29 (0.303)	16.31 (0.305)	0.185 (0.064)
Head Circumference	0 PC	9.85	8.5e-4	0.722 (0.078)	3.52 (0.079)	4.24 (0.080)	0.170 (0.057)
	1 PC	2.79	0.047	0.442 (0.078)	3.78 (0.079)	4.23 (0.079)	0.104 (0.063)
	2 PCs	2.16	0.071	0.393 (0.078)	3.83 (0.079)	4.22 (0.079)	0.093 (0.064)
	5 PCs	1.88	0.085	0.368 (0.078)	3.85 (0.079)	4.22 (0.079)	0.087 (0.064)
	10 PCs	1.93	0.082	0.373 (0.078)	3.85 (0.079)	4.22 (0.079)	0.088 (0.064)
	20 PCs	1.92	0.083	0.372 (0.078)	3.85 (0.079)	4.22 (0.080)	0.088 (0.064)
WaisttoHipRatio	0 PC	13.09	1.5e-4	9.2e-4 (8.6e-5)	3.6e-3 (8.7e-5)	4.5e-3 (8.7e-5)	0.205 (0.061)
	1 PC	4.29	0.019	6.0e-4 (8.6e-5)	3.9e-3 (8.7e-5)	4.5e-3 (8.7e-5)	0.135 (0.067)
	5 PCs	4.11	0.021	5.9e-4 (8.6e-5)	3.9e-3 (8.7e-5)	4.5e-3 (8.7e-5)	0.133 (0.067)
	10 PCs	4.28	0.019	6.1e-4 (8.6e-5)	3.9e-3 (8.7e-5)	4.5e-3 (8.7e-5)	0.136 (0.067)
	20 PCs	3.94	0.024	5.8e-4 (8.6e-5)	3.9e-3 (8.7e-5)	4.5e-3 (8.7e-5)	0.131 (0.066)

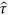
 is the estimated genetic variance, 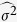 the estimated residual variance, 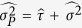 the estimated total variance, and 
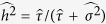
 estimated heritability.

## References

[b1] Galton F. Hereditary genius (Macmillan and Company, 1869).

[b2] Fisher R. The correlation between relatives on the supposition of mendelian inheritance. Trans. R. Soc. Edinb. Earth Sci. 52, 399–433 (1918).

[b3] Falconer D. S. Introduction to quantitative genetics (Oliver & Boyd, 1960).

[b4] Kempthorne O. & Osborne R. H. The interpretation of twin data. Am. J. Hum. Genet. 13, 320 (1961). 13752449 PMC1932084

[b5] Scarr S. Environmental bias in twin studies. Eugenics Q. 15, 34–40 (1968). 10.1080/19485565.1968.9987750 5749030

[b6] Feero W. G., Guttmacher A. E. & Manolio T. A. Genomewide association studies and assessment of the risk of disease. N. Engl. J. Med. 363, 166–176 (2010). 20647212 10.1056/NEJMra0905980

[b7] Donnelly P. Progress and challenges in genome-wide association studies in humans. Nature 456, 728–731 (2008). 19079049 10.1038/nature07631

[b8] Maher B. Personal genomes: The case of the missing heritability. Nature 456, 18–21 (2008). 18987709 10.1038/456018a

[b9] Visscher P. M., Hill W. G. & Wray N. R. Heritability in the genomics era—concepts and misconceptions. Nat. Rev. Genet. 9, 255–266 (2008). 18319743 10.1038/nrg2322

[b10] Zuk O., Hechter E., Sunyaev S. R. & Lander E. S. The mystery of missing heritability: Genetic interactions create phantom heritability. Proc. Natl. Acad. Sci. 109, 1193–1198 (2012). 22223662 10.1073/pnas.1119675109 PMC3268279

[b11] Manolio T. A. . Finding the missing heritability of complex diseases. Nature 461, 747–753 (2009). 19812666 10.1038/nature08494 PMC2831613

[b12] Gusev A. . Quantifying missing heritability at known gwas loci. PLos Genet. 9, e1003993 (2013). 24385918 10.1371/journal.pgen.1003993 PMC3873246

[b13] Lee S. H., Wray N. R., Goddard M. E. & Visscher P. M. Estimating missing heritability for disease from genome-wide association studies. Am. J. Hum. Genet. 88, 294–305 (2011). 21376301 10.1016/j.ajhg.2011.02.002 PMC3059431

[b14] Yang J. . Common SNPs explain a large proportion of the heritability for human height. Nat. Genet. 42, 565–569 (2010). 20562875 10.1038/ng.608 PMC3232052

[b15] Goddard M., Lee H., Yang J., Wray N. & Visscher P. Response to browning and browning. Am. J. Hum. Genet. 89, 193–195 (2011).

[b16] Browning S. R. & Browning B. L. Population structure can inflate SNP-based heritability estimates. Am. J. Hum. Genet. 89, 191–193 (2011). 21763486 10.1016/j.ajhg.2011.05.025 PMC3135810

[b17] Price A. L. . Principal components analysis corrects for stratification in genome-wide association studies. Nat. Genet. 38, 904–909 (2006). 16862161 10.1038/ng1847

[b18] Zhang Y. & Pan W. Principal component regression and linear mixed model in association analysis of structured samples: competitors or complements? Genet. Epidemiol. 39, 149–155 (2015). 25536929 10.1002/gepi.21879 PMC4366301

[b19] Janss L., de Los Campos G., Sheehan N. & Sorensen D. Inferences from genomic models in stratified populations. Genetics 192, 693–704 (2012). 22813891 10.1534/genetics.112.141143 PMC3454890

[b20] Segura V. . An efficient multi-locus mixed-model approach for genome-wide association studies in structured populations. Nat. Genet. 44, 825–830 (2012). 22706313 10.1038/ng.2314 PMC3386481

[b21] Price A. L., Zaitlen N. A., Reich D. & Patterson N. New approaches to population stratification in genome-wide association studies. Nat. Rev. Genet. 11, 459–463 (2010). 20548291 10.1038/nrg2813 PMC2975875

[b22] Group C. S. . Vascular factors and risk of dementia: design of the three-city study and baseline characteristics of the study population. Neuroepidemiology 22, 316 (2003). 14598854 10.1159/000072920

[b23] Novembre J. . Genes mirror geography within Europe. Nature 456, 98–101 (2008). 18758442 10.1038/nature07331 PMC2735096

[b24] Galton F. Regression towards mediocrity in hereditary stature. The Journal of the Anthropological Institute of Great Britain and Ireland 15, 246–263 (1886).

[b25] Allen H. L. . Hundreds of variants clustered in genomic loci and biological pathways affect human height. Nature 467, 832–838 (2010). 20881960 10.1038/nature09410 PMC2955183

[b26] Lanktree M. B. . Meta-analysis of dense genecentric association studies reveals common and uncommon variants associated with height. Am. J. Hum. Genet. 88, 6–18 (2011). 21194676 10.1016/j.ajhg.2010.11.007 PMC3014369

[b27] van der Valk R. J. . A novel common variant in DCST2 is associated with length in early life and height in adulthood. Hum. Mol. Genet. 24, 1155–1168 (2015). 25281659 10.1093/hmg/ddu510 PMC4447786

[b28] Speliotes E. K. . Association analyses of 249,796 individuals reveal 18 new loci associated with body mass index. Nat. Genet. 42, 937–948 (2010). 20935630 10.1038/ng.686 PMC3014648

[b29] Thorleifsson G. . Genome-wide association yields new sequence variants at seven loci that associate with measures of obesity. Nat. Genet. 41, 18–24 (2009). 19079260 10.1038/ng.274

[b30] Loos R. J. Genetic determinants of common obesity and their value in prediction. Best Pract. Res. Clin. Endocrinol. Metab. 26, 211–226 (2012). 22498250 10.1016/j.beem.2011.11.003

[b31] Heid I. M. . Meta-analysis identifies 13 new loci associated with waist-hip ratio and reveals sexual dimorphism in the genetic basis of fat distribution. Nat. Genet. 42, 949–960 (2010). 20935629 10.1038/ng.685 PMC3000924

[b32] Lindgren C. M. . Genome-wide association scan meta-analysis identifies three loci influencing adiposity and fat distribution. PLos Genet. 5, e1000508 (2009). 19557161 10.1371/journal.pgen.1000508 PMC2695778

[b33] Yoneyama S. . Gene-centric meta-analyses for central adiposity traits in up to 57,412 individuals of european descent confirm known loci and reveal several novel associations. Hum. Mol. Genet. 23, 2498–2510 (2014). 24345515 10.1093/hmg/ddt626 PMC3988452

[b34] Stram D. O. & Lee J. W. Variance components testing in the longitudinal mixed effects model. Biometrics 1171–1177 (1994). 7786999

[b35] Yang J. . Genome partitioning of genetic variation for complex traits using common SNPs. Nat. Genet. 43, 519–525 (2011). 21552263 10.1038/ng.823 PMC4295936

[b36] Kumar S. K., Feldman M. W., Rehkopf D. H. & Tuljapurkar S. Limitations of GCTA as a solution to the missing heritability problem. Proc. Natl. Acad. Sci. 113, E61–E70 (2016). 26699465 10.1073/pnas.1520109113 PMC4711841

[b37] Yang J., Lee S. H., Wray N. R., Goddard M. E. & Visscher P. M. Commentary on “Limitations of GCTA as a solution to the missing heritability problem”. *bioRxiv* (Date of access: 04/04/2016) URL http://biorxiv.org/content/early/2016/01/20/036574 (2016).

[b38] Génin E. & Clerget-Darpoux F. The missing heritability paradigm: A dramatic resurgence of the GIGO Syndrome in genetics. Hum. Hered. 79, 10–13 (2015). 10.1159/000370327 25660130

[b39] Lee S. H. & Van Der Werf J. H. An efficient variance component approach implementing an average information REML suitable for combined LD and linkage mapping with a general complex pedigree. Genet. Sel. Evol. 38, 1–19 (2006). 10.1186/1297-9686-38-1-25 PMC2689296 16451790

[b40] Liu D., Ghosh D. & Lin X. Estimation and testing for the effect of a genetic pathway on a disease outcome using logistic kernel machine regression via logistic mixed models. BMC Bioinformatics 9, 292 (2008). 18577223 10.1186/1471-2105-9-292 PMC2483287

[b41] Gilmour A. R., Thompson R. & Cullis B. R. Average information REML: an efficient algorithm for variance parameter estimation in linear mixed models. Biometrics 1440–1450 (1995).

[b42] Lippert C. . FaST linear mixed models for genome-wide association studies. Nat. Methods 8, 833–835 (2011). 21892150 10.1038/nmeth.1681

[b43] Perdry H. & Dandine-Roulland C. Package R ‘gaston’, [version 1.4]. URL https://cran.r-project.org/web/packages/gaston/index.html (2015).

[b44] Yang J., Lee S. H., Goddard M. E. & Visscher P. M. GCTA: a tool for genome-wide complex trait analysis. Am. J. Hum. Genet. 88, 76–82 (2011). 21167468 10.1016/j.ajhg.2010.11.011 PMC3014363

[b45] Anderson C. A. . Data quality control in genetic case-control association studies. Nat. Protoc. 5, 1564–1573 (2010). 21085122 10.1038/nprot.2010.116 PMC3025522

[b46] Lambert J.-C. . Genome-wide association study identifies variants at CLU and CR1 associated with Alzheimer’s disease. Nat. Genet. 41, 1094–1099 (2009). 19734903 10.1038/ng.439

[b47] Purcell S. . Plink: a tool set for whole-genome association and population-based linkage analyses. Am. J. Hum. Genet. 81, 559–575 (2007). 17701901 10.1086/519795 PMC1950838

[b48] Price A. L. . Long-range LD can confound genome scans in admixed populations. Am. J. Hum. Genet. 83, 132 (2008). 18606306 10.1016/j.ajhg.2008.06.005 PMC2443852

[b49] Visscher P. M. . Genome partitioning of genetic variation for height from 11,214 sibling pairs. Am. J. Hum. Genet. 81, 1104–1110 (2007). 17924350 10.1086/522934 PMC2265649

[b50] Macgregor S., Cornes B. K., Martin N. G. & Visscher P. M. Bias, precision and heritability of self-reported and clinically measured height in Australian twins. Hum. Genet. 120, 571–580 (2006). 16933140 10.1007/s00439-006-0240-z

[b51] Silventoinen K. . Heritability of adult body height: a comparative study of twin cohorts in eight countries. Twin Res. 6, 399–408 (2003). 14624724 10.1375/136905203770326402

[b52] Polderman T. J. . Meta-analysis of the heritability of human traits based on fifty years of twin studies. Nat. Genet. 47, 702–709 (2015). 25985137 10.1038/ng.3285

[b53] Chen X. . Dominant genetic variation and missing heritability for human complex traits: Insights from twin versus genome-wide common snp models. Am. J. Hum. Genet. 97, 708–714 (2015). 26544805 10.1016/j.ajhg.2015.10.004 PMC4667127

[b54] Visscher P. M., Yang J. & Goddard M. E. A commentary on ‘common SNPs explain a large proportion of the heritability for human height’ by Yang *et al*. Twin Res. Hum. Genet. 13, 517–524 (2010). 21142928 10.1375/twin.13.6.517

[b55] Yang J. . Genetic variance estimation with imputed variants finds negligible missing heritability for human height and body mass index. Nat. Genet. 47, 1114–1120 (2015). 26323059 10.1038/ng.3390 PMC4589513

[b56] Speed D., Hemani G., Johnson M. R. & Balding D. J. Improved heritability estimation from genome-wide SNPs. Am. J. Hum. Genet. 91, 1011–1021 (2012). 23217325 10.1016/j.ajhg.2012.10.010 PMC3516604

[b57] Yang J. . Genome-wide genetic homogeneity between sexes and populations for human height and body mass index. Hum. Mol. Genet. ddv443 (2015). 10.1093/hmg/ddv443 26494901

[b58] Elks C. E. . Variability in the heritability of body mass index: a systematic review and meta-regression. Front. Endocrinol. 3, 29 (2012). 10.3389/fendo.2012.00029 PMC3355836 22645519

[b59] Smit D. J. . Heritability of head size in Dutch and Australian twin families at ages 0–50 years. Twin Res. Hum. Genet. 13, 370–380 (2010). 20707707 10.1375/twin.13.4.370

[b60] Ermakov S., Kobyliansky E. & Livshits G. Quantitative genetic study of head size related phenotypes in ethnically homogeneous chuvasha pedigrees. Ann. Hum. Biol. 32, 585–598 (2005). 16316915 10.1080/03014460500247972

