# Supplementary material for: Accuracy of heritability estimations in presence of hidden population stratification
Source: Sci Rep. 2016 May 25;6:26471. doi: 10.1038/srep26471 (PMC4879529; doi:10.1038/srep26471)
Supplement: Supplementary Information [file srep26471-s1.pdf]

# Accuracy of heritability estimations in presence of hidden population stratification

Claire Dandine-Roulland, Céline Bellenguez, Stéphanie Debette, Philippe Amouyel, Emmanuelle Génin, and Hervé Perdry

# Supplementary Information 1

## Supplementary figures

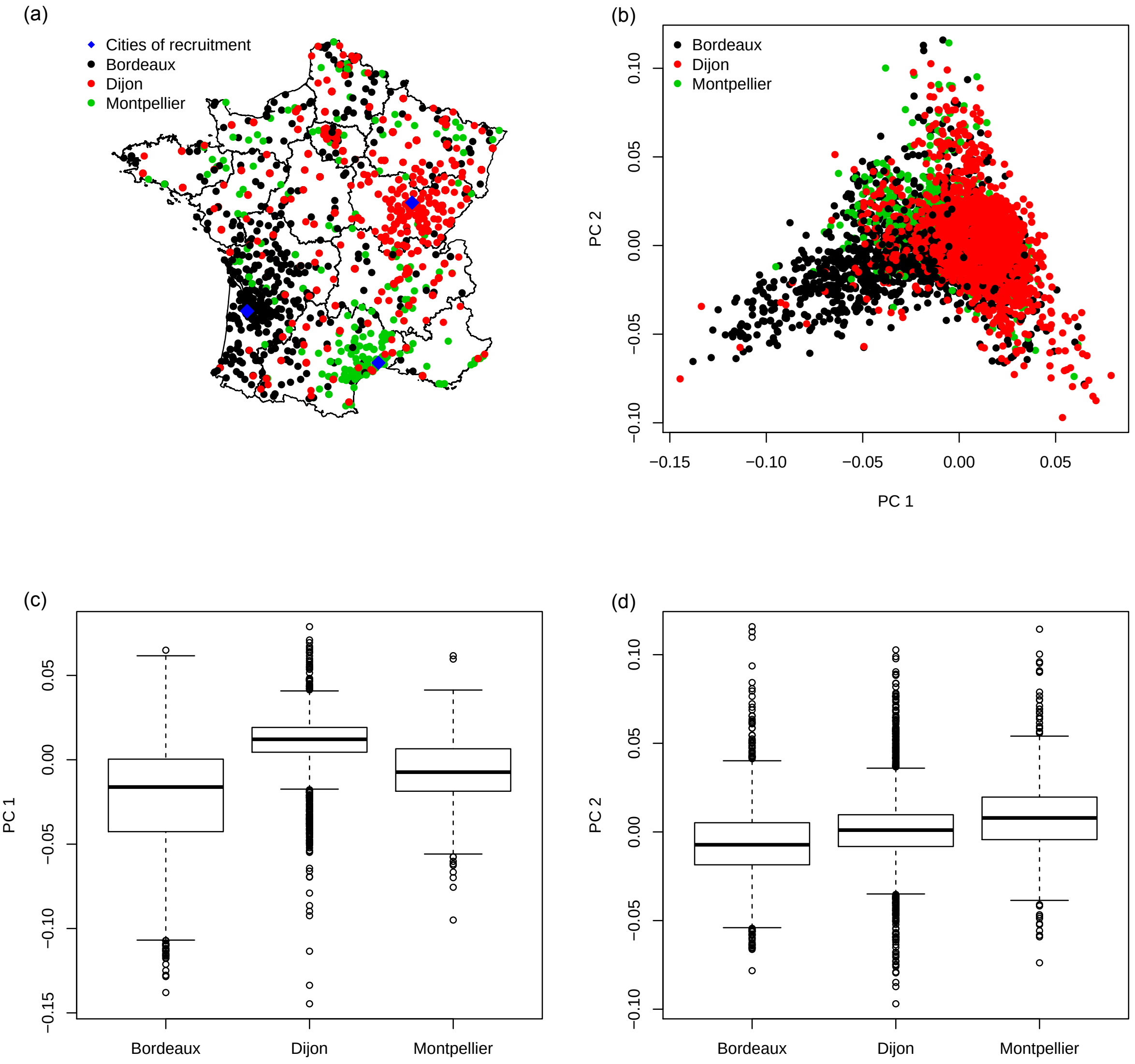


Figure S1: Distribution of geographical coordinates and first two PCs depending of recruitment center, Bordeaux (1499 individuals), Dijon (3676 individuals) and Montpellier (618 individuals). (a) Plot of geographical coordinates on map of France. The map was drawn using the R package 'rgdal' (version 1.1-3, `https://cran.r-project.org/web/packages/rgdal/index.html`) with border coordinates from ©OpenStreetMap contributors, license ODbL `http://www.openstreetmap.org/copyright/en`. (b) Plot of first two PCs. (c) Boxplots of (c) first and (d) second PC.

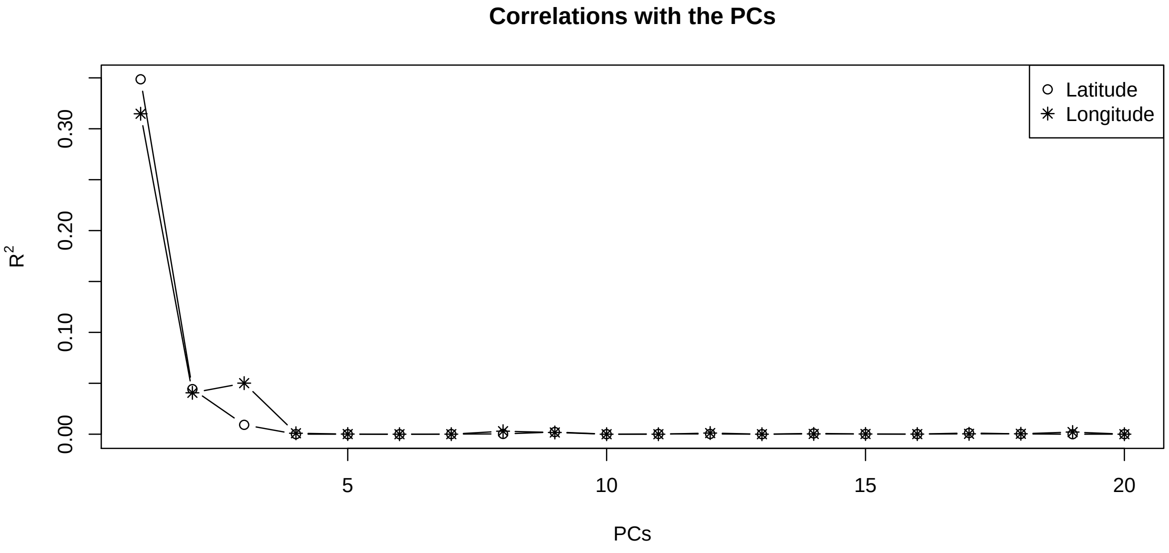


Figure S2: Correlation ($R^2$) of the geographical coordinates with the first 20 PCs.

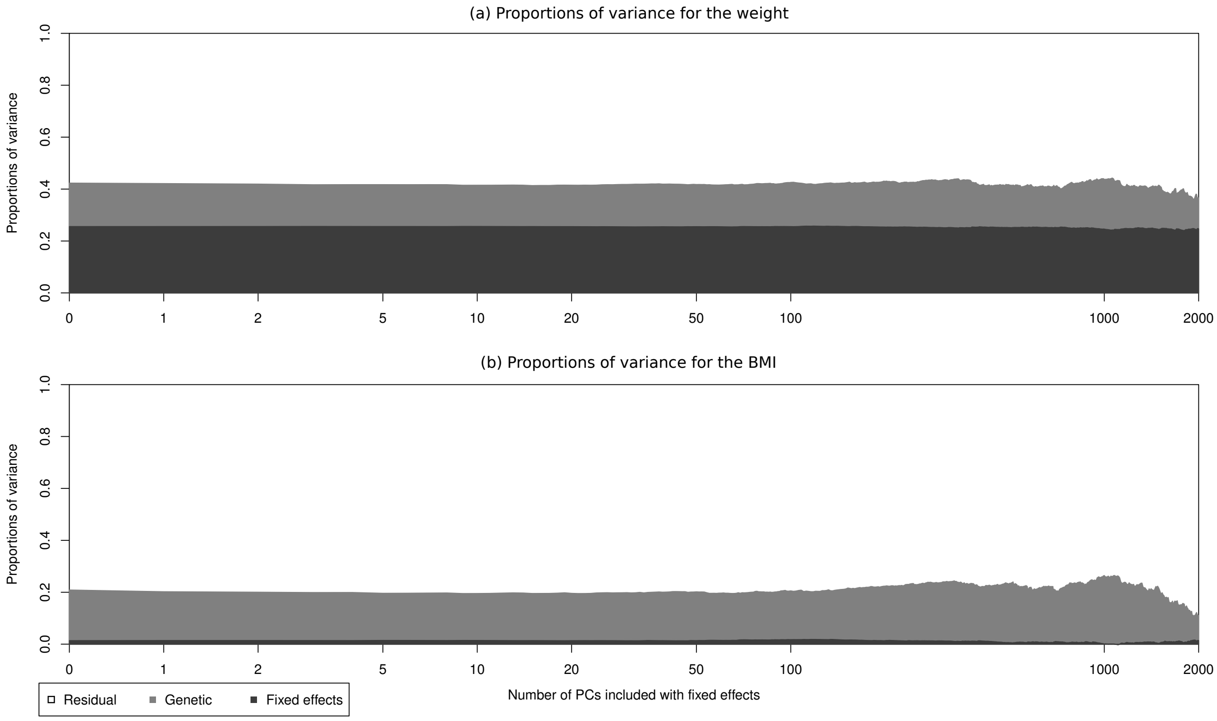


Figure S3: Estimated proportion of variance for (a) weight and (b) BMI, depending on the number of PCs included in the model (log-scale). The white, light gray and dark gray are respectively the residual, genetic and fixed effects variances.

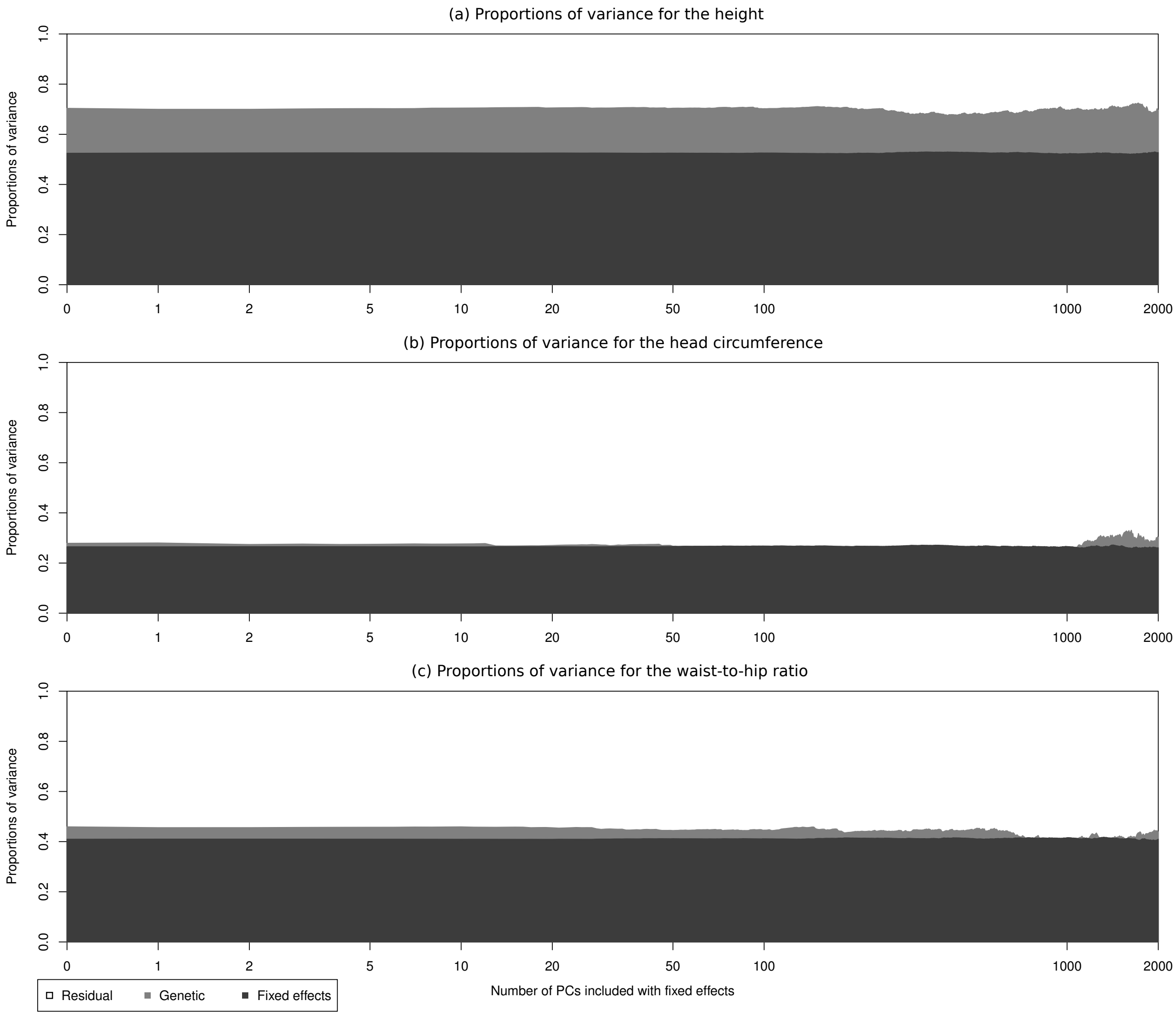


Figure S4: Estimated proportion of variance for (a) height, (b) head circumference, and (c) waist-to-hip-ratio, including longitude and latitude as covariates and depending on the number of PCs included in the model (log-scale). The white, light gray and dark gray are respectively the residual, genetic and fixed effects variances.

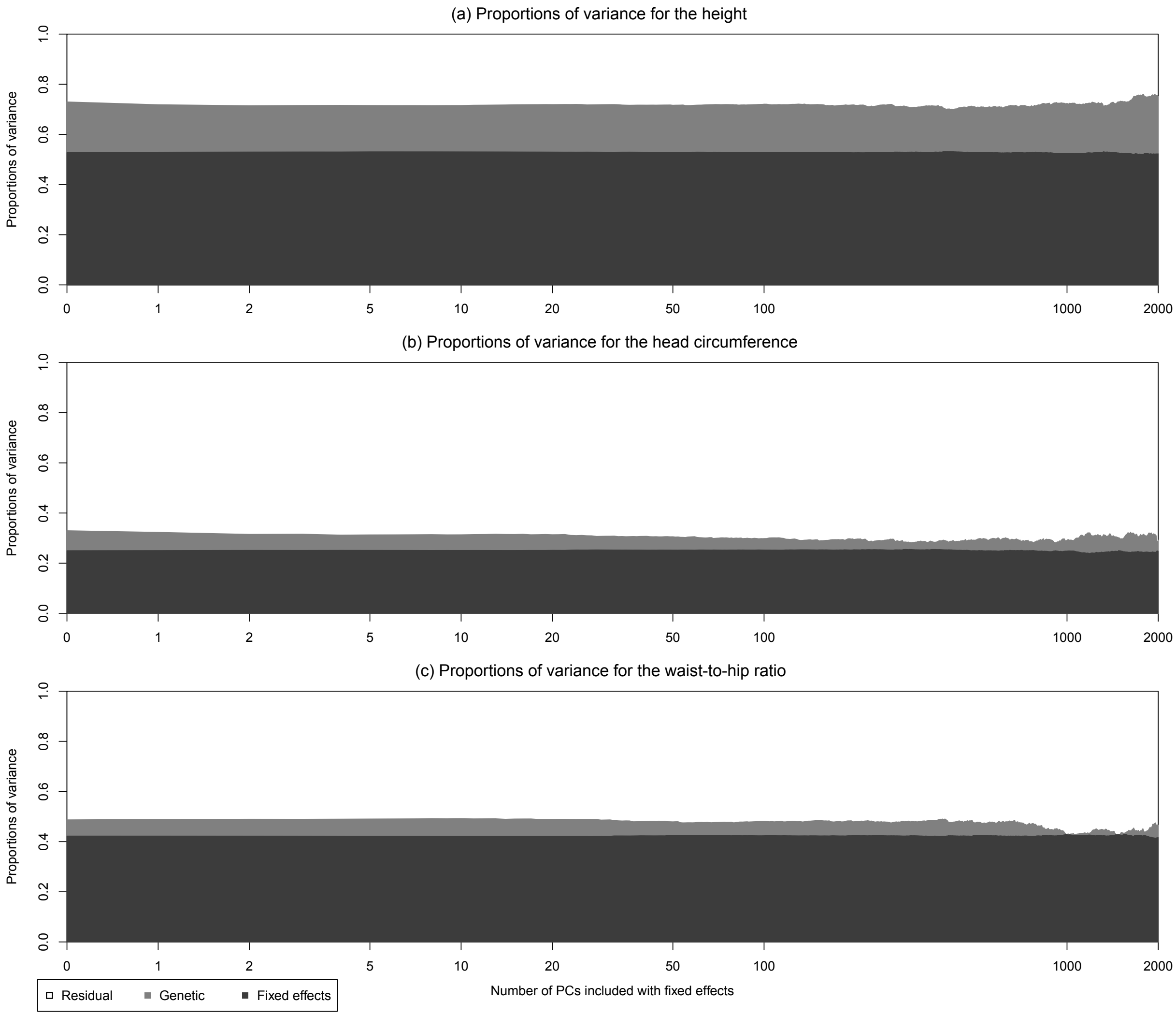


Figure S5: Estimated proportion of variance for (a) height, (b) head circumference, and (c) waist-to-hip-ratio, including centers as covariates and depending on the number of PCs included in the model (log-scale). The white, light gray and dark gray are respectively the residual, genetic and fixed effects variances.

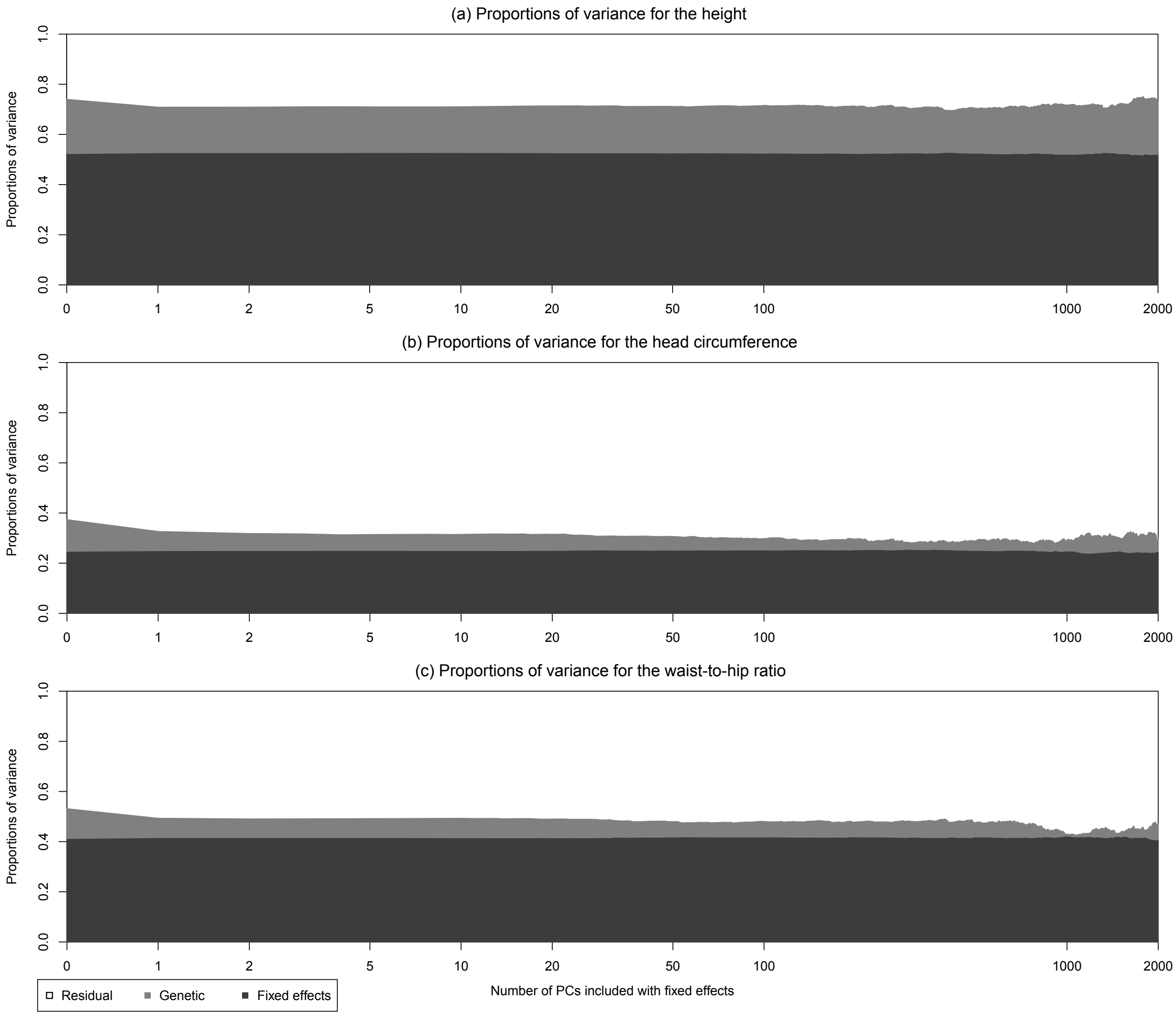


Figure S6: Estimated proportion of variance for (a) height, (b) head circumference, and (c) waist-to-hip-ratio, considering only individuals recruited in Dijon and depending on the number of PCs included in the model (log-scale). The white, light gray and dark gray are respectively the residual, genetic and fixed effects variances.

# Supplementary tables

| Phenotype | | LRT | $p$-value | $\widehat{\tau}$ (se) | $\widehat{\sigma^2}$ (se) | $\widehat{\sigma^2_P}$ (se) | $\widehat{h^2}$ (se) |
|---|---|---|---|---|---|---|---|
| Height | 0 PC | 30.05 | 2.1e-8 | 14.09 (0.737) | 23.29 (0.712) | 37.38 (0.715) | 0.377 (0.070) |
| | 1 PC | 27.98 | 6.1e-8 | 13.72 (0.736) | 23.61 (0.715) | 37.33 (0.714) | 0.368 (0.071) |
| | 5 PCs | 28.59 | 4.5e-8 | 13.89 (0.734) | 23.39 (0.712) | 37.28 (0.706) | 0.373 (0.071) |
| | 10 PCs | 29.34 | 3.0e-8 | 14.08 (0.736) | 23.22 (0.715) | 37.30 (0.712) | 0.378 (0.071) |
| Head | 0 PC | 0.07 | 0.39 | 0.077 (0.078) | 3.95 (0.080) | 4.03 (0.080) | 0.019 (0.071) |
| Circumference | 1 PC | 0.09 | 0.38 | 0.084 (0.078) | 3.95 (0.080) | 4.03 (0.080) | 0.021 (0.071) |
| | 5 PCs | 0.03 | 0.43 | 0.053 (0.078) | 3.98 (0.080) | 4.03 (0.080) | 0.013 (0.071) |
| | 10 PCs | 0.05 | 0.41 | 0.064 (0.078) | 3.97 (0.080) | 4.03 (0.080) | 0.016 (0.071) |
| Waist | 0 PC | 1.35 | 0.12 | 3.8e-4 (9.1e-5) | 4.2e-3 (9.3e-5) | 4.5e-3 (9.3e-5) | 0.084 (0.071) |
| to | 1 PC | 1.13 | 0.14 | 3.5e-4 (9.1e-5) | 4.2e-3 (9.3e-5) | 4.5e-3 (9.3e-5) | 0.078 (0.072) |
| Hip | 5 PCs | 1.23 | 0.13 | 3.7e-4 (9.1e-5) | 4.2e-3 (9.3e-5) | 4.5e-3 (9.3e-5) | 0.081 (0.076) |
| Ratio | 10 PCs | 1.32 | 0.13 | 3.8e-4 (9.1e-5) | 4.2e-3 (9.3e-5) | 4.5e-3 (9.3e-5) | 0.084 (0.073) |

Table S1: Model parameter estimates for three anthropometric phenotypes and their standard error when the geographical coordinates are included in the model together with the sex, age, and a variable number of PCs. The table includes also likelihood ratio test statistics (LRT) to test significance of heritability and their $p$-values. $\widehat{\tau}$ is the estimated genetic variance, $\widehat{\sigma^2}$ the estimated residual variance, $\widehat{\sigma^2_P} = \widehat{\tau} + \widehat{\sigma^2}$ the estimated total variance, and $\widehat{h^2} = \widehat{\tau}/(\widehat{\tau} + \widehat{\sigma^2})$ estimated heritability.

| Phenotype | | LRT | $p$-value | $\widehat{\tau}$ (se) | $\widehat{\sigma^2}$ (se) | $\widehat{\sigma_P^2}$ (se) | $\widehat{h^2}$ (se) |
|---|---|---|---|---|---|---|---|
| Height | 0 PC | 49.40 | 1.0e-12 | 16.06 (0.707) | 21.47 (0.682) | 37.53 (0.676) | 0.428 (0.063) |
| | 1 PC | 39.82 | 1.4e-10 | 15.05 (0.705) | 22.37 (0.682) | 37.42 (0.679) | 0.402 (0.065) |
| | 5 PCs | 37.45 | 4.7e-10 | 14.69 (0.702) | 22.60 (0.681) | 37.29 (0.676) | 0.394 (0.065) |
| | 10 PCs | 37.36 | 4.9e-10 | 14.71 (0.703) | 22.60 (0.681) | 37.31 (0.677) | 0.394 (0.066) |
| Head | 0 PC | 2.99 | 0.042 | 0.446 (0.077) | 3.76 (0.079) | 4.21 (0.079) | 0.106 (0.062) |
| Circumference | 1 PC | 2.39 | 0.061 | 0.407 (0.077) | 3.80 (0.079) | 4.21 (0.079) | 0.097 (0.063) |
| | 5 PCs | 1.69 | 0.097 | 0.347 (0.077) | 3.85 (0.079) | 4.20 (0.079) | 0.083 (0.064) |
| | 10 PCs | 1.73 | 0.094 | 0.351 (0.077) | 3.85 (0.079) | 4.20 (0.079) | 0.084 (0.064) |
| Waist | 0 PC | 2.94 | 0.043 | 4.9e-4 (8.4e-5) | 3.9e-3 (8.5e-5) | 4.4e-3 (8.6e-5) | 0.112 (0.067) |
| to | 1 PC | 3.10 | 0.039 | 5.1e-4 (8.4e-5) | 3.9e-3 (8.5e-5) | 4.4e-3 (8.6e-5) | 0.115 (0.066) |
| Hip | 5 PCs | 3.30 | 0.035 | 5.2e-4 (8.4e-5) | 3.9e-3 (8.6e-5) | 4.4e-3 (8.6e-5) | 0.118 (0.066) |
| Ratio | 10 PCs | 3.44 | 0.032 | 5.3e-4 (8.4e-5) | 3.9e-3 (8.6e-5) | 4.4e-3 (8.6e-5) | 0.121 (0.067) |

Table S2: Model parameter estimates for three anthropometric phenotypes and their standard error when the centers are included in the model together with the sex, age, and a variable number of PCs. The table includes also likelihood ratio test statistics (LRT) to test significance of heritability and their $p$-values. $\widehat{\tau}$ is the estimated genetic variance, $\widehat{\sigma^2}$ the estimated residual variance, $\widehat{\sigma_P^2} = \widehat{\tau} + \widehat{\sigma^2}$ the estimated total variance, and $\widehat{h^2} = \widehat{\tau}/(\widehat{\tau} + \widehat{\sigma^2})$ estimated heritability.

| Phenotype | | LRT | $p$-value | $\widehat{\tau}$ (se) | $\widehat{\sigma^2}$ (se) | $\widehat{\sigma_P^2}$ (se) | $\widehat{h^2}$ (se) |
|---|---|---|---|---|---|---|---|
| Height | 0 PC | 68.80 | 5.6e-17 | 17.48 (0.718) | 20.64 (0.690) | 38.13 (0.687) | 0.459 (0.061) |
| | 1 PC | 36.32 | 8.4e-10 | 14.70 (0.713) | 23.13 (0.691) | 37.83 (0.686) | 0.389 (0.066) |
| | 5 PCs | 36.56 | 7.4e-10 | 14.74 (0.711) | 23.03 (0.689) | 37.77 (0.685) | 0.390 (0.066) |
| | 10 PCs | 36.60 | 7.3e-10 | 14.77 (0.713) | 23.01 (0.690) | 37.78 (0.686) | 0.391 (0.066) |
| Head | 0 PC | 10.00 | 7.8e-4 | 0.729 (0.078) | 3.52 (0.079) | 4.24 (0.080) | 0.172 (0.058) |
| Circumference | 1 PC | 2.88 | 0.045 | 0.449 (0.078) | 3.78 (0.079) | 4.23 (0.080) | 0.106 (0.063) |
| | 5 PCs | 1.96 | 0.081 | 0.376 (0.078) | 3.85 (0.079) | 4.22 (0.079) | 0.089 (0.064) |
| | 10 PCs | 2.01 | 0.078 | 0.381 (0.078) | 3.84 (0.079) | 4.22 (0.080) | 0.090 (0.064) |
| Waist | 0 PC | 13.33 | 1.3e-4 | 9.3e-4 (8.6e-5) | 3.6e-3 (8.7e-5) | 4.5e-3 (8.7e-5) | 0.207 (0.062) |
| to | 1 PC | 4.45 | 0.017 | 6.2e-4 (8.6e-5) | 3.9e-3 (8.7e-5) | 4.5e-3 (8.7e-5) | 0.138 (0.067) |
| Hip | 5 PCs | 4.26 | 0.020 | 6.1e-4 (8.6e-5) | 3.9e-3 (8.7e-5) | 4.5e-3 (8.7e-5) | 0.135 (0.067) |
| Ratio | 10 PCs | 4.44 | 0.018 | 6.2e-4 (8.6e-5) | 3.9e-3 (8.7e-5) | 4.5e-3 (8.7e-5) | 0.138 (0.067) |

Table S3: Model parameter estimates for three anthropometric phenotypes and their standard error when only individuals recruited in Dijon are considered. The sex, age, and a variable number of PCs are included in the model. The table includes also likelihood ratio test statistics (LRT) to test significance of heritability and their $p$-values. $\widehat{\tau}$ is the estimated genetic variance, $\widehat{\sigma^2}$ the estimated residual variance, $\widehat{\sigma_P^2} = \widehat{\tau} + \widehat{\sigma^2}$ the estimated total variance, and $\widehat{h^2} = \widehat{\tau}/(\widehat{\tau} + \widehat{\sigma^2})$ estimated heritability.

| Phenotype | PC number | Intercept | $P$-value | slope | $P$-value |
|---|---|---|---|---|---|
| Longitude | 0 PC | 0.192 | **8.0e-13** | 1.0e-3 | **1.4e-10** |
| | 5 PCs | 2.5e-3 | 0.53 | 9.5e-5 | **2.6e-3** |
| | 10 PCs | 1.6e-3 | 0.69 | 8.6e-5 | **6.5e-3** |
| | 20 PCs | 1.3e-3 | 0.72 | 7.9e-5 | **5.5e-3** |
| | 50 PCs | 1.8e-3 | 0.62 | 6.8e-5 | **0.013** |
| | 100 PCs | 2.7e-3 | 0.49 | 4.8e-5 | 0.086 |
| | 1000 PCs | 4.5e-4 | 0.87 | 8.5e-5 | 0.65 |
| Latitude | 0 PC | 0.185 | **3.2e-13** | 8.9e-4 | **2.8e-10** |
| | 5 PCs | 4.1e-3 | 0.14 | 4.5e-5 | **0.025** |
| | 10 PCs | 4.3e-3 | 0.11 | 3.9e-5 | **0.041** |
| | 20 PCs | 4.7e-3 | 0.086 | 2.9e-5 | 0.12 |
| | 50 PCs | 4.5e-3 | 0.097 | 2.5e-5 | 0.17 |
| | 100 PCs | 3.1e-3 | 0.17 | 2.3e-5 | 0.15 |
| Height | 0 PC | 1.4e-3 | 0.46 | 6.8e-5 | **3.9e-5** |
| | 1 PC | -1.3e-5 | 0.99 | 6.9e-6 | 0.44 |
| | 2 PCs | -6.1e-5 | 0.96 | 6.7e-6 | 0.43 |
| Weight | 0 PC | 1.8e-4 | 0.98 | 4.0e-6 | 0.51 |
| | 1 PC | 3.1e-4 | 0.69 | 3.9e-7 | 0.94 |
| | 2 PCs | 2.3e-4 | 0.75 | 4.3e-7 | 0.93 |
| BMI | 0 PC | -1.1e-5 | 0.99 | 4.0e-6 | 0.50 |
| | 1 PC | -5.4e-4 | 0.52 | 4.1e-6 | 0.48 |
| | 2 PCs | -5.5e-4 | 0.50 | 4.0e-6 | 0.48 |
| Head | 0 PC | 1.2e-5 | 0.99 | 2.0e-5 | **6.8e-3** |
| Circumference | 1 PC | 6.6e-4 | 0.14 | -4.5e-6 | 0.15 |
| | 2 PCs | 6.4e-4 | 0.15 | -5.3e-6 | 0.089 |
| Waist | 0 PC | -1.8e-4 | 0.90 | 3.7e-5 | **1.6e-3** |
| to Hip | 1 PC | -5.1e-4 | 0.52 | 1.1e-5 | 0.053 |
| Ratio | 2 PCs | -4.1e-4 | 0.58 | 9.6e-6 | 0.075 |

Table S4: Regression results of the difference between heritability estimates for one chromosome one at a time vs all together on the chromosome length (in mega-bases). A positive intercept is interpreted as proving the presence of cryptic relatedness. A positive slope is interpreted a proving the presence of population stratification.

| Trait | $R^2$ | $p$-value |
|---|---|---|
| Height | 4.0e-3 | 2.9e-5 |
| Weight | 1.3e-4 | 0.70 |
| BMI | 3.4e-3 | 1.3e-4 |
| Head circumference | 6.9e-3 | 1.9e-8 |
| Waist to Hip Ratio | 5.2e-3 | 3.7e-6 |

Table S5: Coefficient of determination of the anthropometric phenotypes by the geographical coordinates, and the associated $p$-value.

# Supplementary Methods

## Variance components estimation

All analysis have been done with R package Gaston.[47] Most results have been double checked using GCTA.[68]

An example of command line to fit a linear mixed model with Gaston, using the "diagonalization trick", is

```
K <- GRM(x)
eigenK <- eigen(K)
fit <- lmm.diago(Y, X, eigenK)
```

The first line calculates the kinship matrix $K$, $x$ being the matrix of all genotypes. The second line calculates the eigen decomposition of $K$ and the last line fits the linear mixed model with $Y$ the phenotype vector and $X$ the matrix of covariates (possibly including PCs).

With GCTA, the same analysis is run by

```
./gcta64 --make-grm-gz --bfile data --out kinship
./gcta64 --reml --reml-est-fix --pheno pheno.phen --grm-gz kinship
         --qcovar X.qcovar --out result
```

The first line calculates the kinship matrix from the genotypes in the files `data.bed`, `data.fam` and `data.bim`. The second line fits the linear mixed model, `pheno.phen` being the file of phenotypes and `X.qcovar` the file of quantitative covariates.

## Unbiased estimation of variance explained by covariates with fixed effects

We consider the linear mixed model

$$Y = X\alpha + Zu + \varepsilon \tag{2}$$

as described in the main text, except that the matrix $X$ can here contain some PCs to be included as fixed effects.

The empirical variance of the components of the phenotype vector $Y$ is

$$\text{ev}(Y) = \frac{1}{n-1}\left(Y'Y - \frac{1}{n}\left(1_n'Y\right)^2\right) \tag{3}$$

Define a linear form $\Psi : \mathbb{R}^{n\times n} \to \mathbb{R}$ by

$$\Psi(A) = \frac{1}{n-1}\left(\text{tr}(A) - \frac{1}{n}1_n' A 1_n\right). \tag{4}$$

We have $\text{ev}(Y) = \Psi(YY')$ for any $Y \in \mathbb{R}^n$.

The linearity of $\Psi$ implies that

$$\begin{aligned} E(\text{ev}(Y)) &= E(\Psi(YY')) \\ &= \Psi(E(YY')) \\ &= \Psi(E(Y)E(Y)' + \text{Var}(Y)) \\ &= \text{ev}(E(Y)) + \Psi(\text{Var}(Y)) \end{aligned} \tag{5}$$

So in our case, with $E(Y) = X\alpha$ and $\text{Var}(Y) = K\tau + \sigma^2 = V$, we have

$$E\left(\text{ev}(Y)\right) = \text{ev}(X\alpha) + \Psi(V). \tag{6}$$

The term $\text{ev}(X\alpha)$ in (6) is the variance due to the factors in $X$. The term $\Psi(V)$ is the variance due to the various random terms (genetic and residual variance). By linearity of $\Psi$, we have

$$\Psi(V) = \tau\Psi(K) + \sigma^2\Psi(I_n) = \tau\Psi(K) + \sigma^2.$$

The matrix $K$ has terms close to $1$ on its diagonal, and other terms are close to $0$, so we have $\Psi(K) \simeq 1$ and

$$E\left(\text{ev}(Y)\right) \simeq \text{ev}(X\alpha) + \sigma^2 + \tau.$$

The REML provides unbiased estimates of $\sigma^2$ and $\tau$.

However, substituting $\widehat{\alpha}$ to $\alpha$ in $\text{ev}(X\alpha)$. leads to a biased estimate:

$$\begin{aligned} E\left(\text{ev}\left(X\widehat{\alpha}\right)\right) &= \text{ev}(X\alpha) + \Psi(\text{Var}(X\widehat{\alpha})) \\ &= \text{ev}(X\alpha) + \Psi\left(X\text{Var}\left(\widehat{\alpha}\right)X'\right) \\ &= \text{ev}(X\alpha) + \Psi\left(X\left(X'V^{-1}X\right)^{-1}X'\right). \end{aligned}$$

Hence, an unbiased estimator of $\text{ev}(X\alpha)$ is given by

$$\text{ev}(X\widehat{\alpha}) - \Psi\left(X\left(X'V^{-1}X\right)^{-1}X'\right).$$

# Accuracy of heritability estimations in presence of hidden population stratification

Claire Dandine-Roulland, Céline Bellenguez, Stéphanie Debette, Philippe Amouyel, Emmanuelle Génin, and Hervé Perdry

## Supplementary Information 2

Here we show the results of the analyses described in the main text, when the PCs included in the model as fixed effect for population stratification correction are computed on the whole data instead of LD-pruned data.

Figure S7 shows the decomposition of variance of the geographical coordinates, for $p$ varying from $0$ to $2000$. Tables S6 and S7 give precise figures, in particular heritability estimates. The results are marginally different from the ones described in the main text, but this does not alter our conclusions: including a handful of PCs with a fixed effect is not sufficient to correct for population stratification.

The analysis of anthropometric phenotypes leads to similar conclusion than previously: weight and BMI heritability estimates are not affected by the inclusion of PCs (table S8), while the heritability of height, head circumference, and waist-to-hip ratio drops with the first PC included, and is no more affected by the inclusion of additional PCs (table S8 and figure S8).

## Supplementary figures

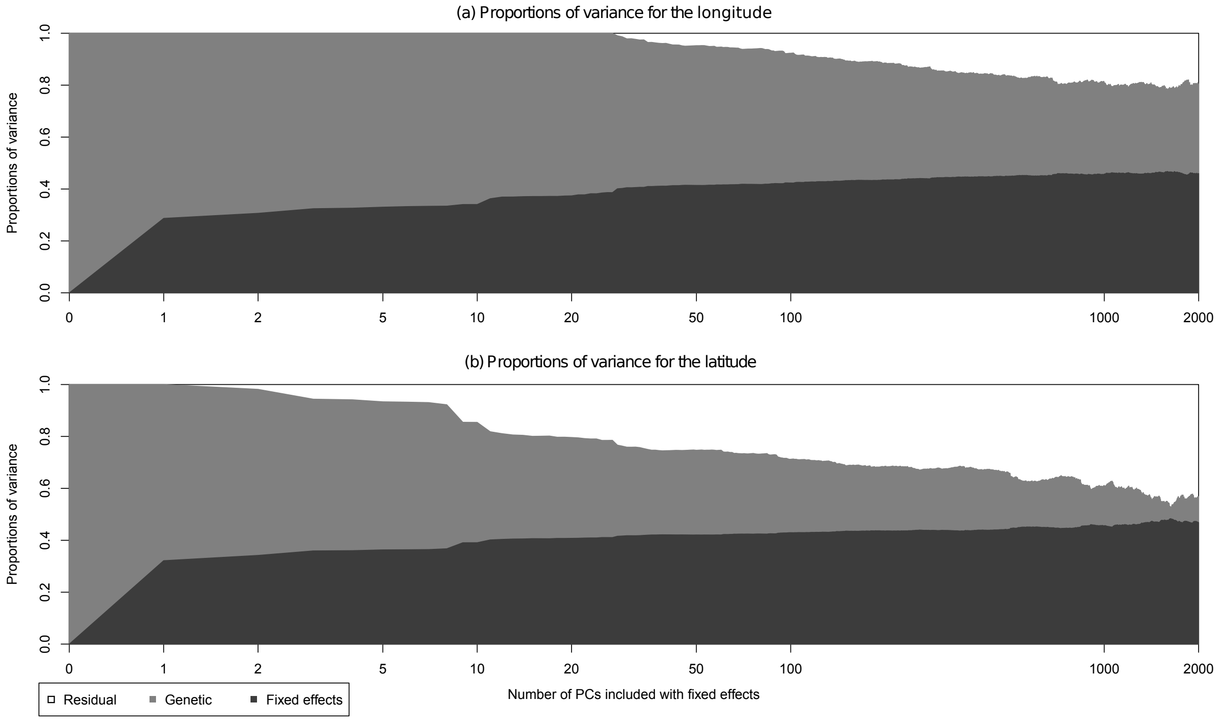


Figure S7: Estimated proportion of variance for the geographical coordinates, depending on the number of PCs included in the model (log-scale). Included PCs are computed using all SNPs. The white, light gray and dark gray are respectively the residual, genetic and fixed effects variances.

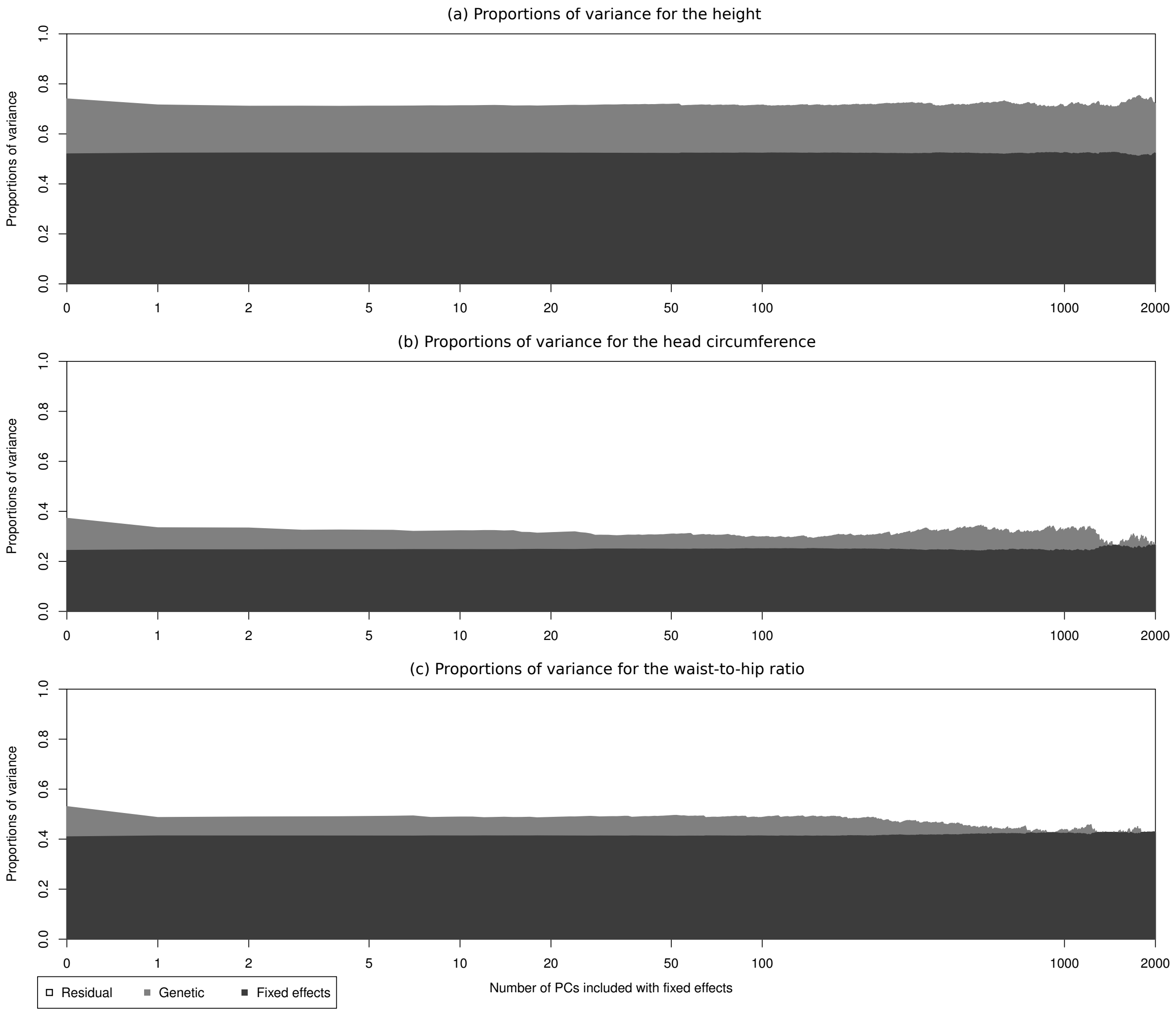


Figure S8: Estimated proportion of variance for the (a) height, (b) head circumference, and (c) waist-to-hip-ratio, depending on the number of PCs included in the model (log-scale). Included PCs are computed using all SNPs. The white, light gray and dark gray are respectively the residual, genetic and fixed effects variances.

## Supplementary tables

| | LRT | $p$-value | $\widehat{\tau}$ (se) | $\widehat{\sigma^2}$ (se) | $\widehat{\sigma^2_P}$ (se) | $\widehat{h^2}$ (se) |
|---|---|---|---|---|---|---|
| 0 PC | 1892.63 | $<$1e-40 | 4.34 (0.085) | 2.9e-4 (0.089) | 4.34 (0.084) | 1.000 |
| 1 PC | 822.44 | $<$1e-40 | 3.90 (0.076) | 2.6e-4 (0.072) | 3.90 (0.076) | 1.000 |
| 2 PCs | 708.77 | $<$1e-40 | 3.86 (0.075) | 2.6e-4 (0.070) | 3.86 (0.075) | 1.000 |
| 3 PCs | 600.64 | $<$1e-40 | 3.82 (0.075) | 2.5e-4 (0.069) | 3.82 (0.074) | 1.000 |
| 4 PCs | 598.75 | $<$1e-40 | 3.82 (0.074) | 2.5e-4 (0.069) | 3.82 (0.074) | 1.000 |
| 5 PCs | 570.04 | $<$1e-40 | 3.80 (0.074) | 2.5e-4 (0.068) | 3.80 (0.074) | 1.000 |
| 10 PCs | 524.18 | $<$1e-40 | 3.77 (0.074) | 2.5e-4 (0.067) | 3.77 (0.073) | 1.000 |
| 20 PCs | 377.77 | $<$1e-40 | 3.65 (0.071) | 2.4e-4 (0.063) | 3.65 (0.071) | 1.000 |
| 50 PCs | 207.02 | $<$1e-40 | 3.19 (0.068) | 0.28 (0.060) | 3.47 (0.068) | 0.919 (0.061) |
| 100 PCs | 168.31 | 8.7e-39 | 2.96 (0.068) | 0.46 (0.060) | 3.42 (0.067) | 0.867 (0.064) |
| 500 PCs | 69.56 | 3.7e-17 | 2.29 (0.069) | 0.98 (0.061) | 3.27 (0.067) | 0.701 (0.079) |
| 1000 PCs | 38.23 | 3.2e-10 | 2.07 (0.073) | 1.14 (0.063) | 3.22 (0.070) | 0.645 (0.095) |
| 2000 PCs | 15.93 | 3.3e-5 | 2.02 (0.087) | 1.18 (0.068) | 3.20 (0.079) | 0.632 (0.136) |

Table S6: Model parameter estimates for the longitude and their standard error, depending on the number of PCs included in the model, likelihood ratio test statistics (LRT) to test significance of heritability and their $p$-values. $\widehat{\tau}$ is the estimated genetic variance, $\widehat{\sigma^2}$ the estimated residual variance, $\widehat{\sigma^2_P} = \widehat{\tau} + \widehat{\sigma^2}$ the estimated total variance, and $\widehat{h^2} = \widehat{\tau}/(\widehat{\tau} + \widehat{\sigma^2})$ estimated heritability.

| | LRT | $p$-value | $\widehat{\tau}$ (se) | $\widehat{\sigma^2}$ (se) | $\widehat{\sigma^2_P}$ (se) | $\widehat{h^2}$ (se) |
|---|---|---|---|---|---|---|
| 0 PC | 1854.15 | $<$1e-40 | 2.05 (0.040) | 1.4e-4 (0.039) | 2.05 (0.040) | 1.000 |
| 1 PC | 558.69 | $<$1e-40 | 1.81 (0.035) | 1.2e-4 (0.031) | 1.81 (0.035) | 1.000 |
| 2 PCs | 424.64 | $<$1e-40 | 1.74 (0.035) | 0.05 (0.030) | 1.79 (0.035) | 0.972 (0.050) |
| 3 PCs | 312.82 | $<$1e-40 | 1.61 (0.035) | 0.15 (0.030) | 1.76 (0.034) | 0.912 (0.053) |
| 4 PCs | 307.45 | $<$1e-40 | 1.60 (0.035) | 0.16 (0.030) | 1.76 (0.034) | 0.908 (0.053) |
| 5 PCs | 292.00 | $<$1e-40 | 1.57 (0.034) | 0.18 (0.030) | 1.75 (0.034) | 0.896 (0.054) |
| 10 PCs | 177.38 | $<$1e-40 | 1.28 (0.033) | 0.40 (0.030) | 1.69 (0.033) | 0.761 (0.058) |
| 20 PCs | 114.47 | 5.1e-27 | 1.08 (0.033) | 0.57 (0.030) | 1.65 (0.032) | 0.656 (0.062) |
| 50 PCs | 74.84 | 2.5e-18 | 0.91 (0.032) | 0.70 (0.030) | 1.61 (0.032) | 0.563 (0.065) |
| 100 PCs | 52.25 | 2.4e-13 | 0.79 (0.032) | 0.80 (0.030) | 1.59 (0.031) | 0.496 (0.068) |
| 500 PCs | 21.08 | 2.2e-6 | 0.60 (0.033) | 0.95 (0.031) | 1.55 (0.032) | 0.387 (0.082) |
| 1000 PCs | 7.10 | 3.9e-3 | 0.43 (0.036) | 1.08 (0.032) | 1.51 (0.033) | 0.286 (0.104) |
| 2000 PCs | 1.18 | 0.139 | 0.28 (0.044) | 1.20 (0.035) | 1.48 (0.037) | 0.189 (0.167) |

Table S7: Model parameter estimates for the latitude and their standard error, depending on the number of PCs included in the model, likelihood ratio test statistics (LRT) to test significance of heritability and their $p$-values. $\widehat{\tau}$ is the estimated genetic variance, $\widehat{\sigma^2}$ the estimated residual variance, $\widehat{\sigma^2_P} = \widehat{\tau} + \widehat{\sigma^2}$ the estimated total variance, and $\widehat{h^2} = \widehat{\tau}/(\widehat{\tau} + \widehat{\sigma^2})$ estimated heritability.

| Phenotype | | LRT | $p$-value | $\widehat{\tau}$ (se) | $\widehat{\sigma^2}$ (se) | $\widehat{\sigma^2_P}$ (se) | $\widehat{h^2}$ (se) |
|---|---|---|---|---|---|---|---|
| Height | 0 PC | 68.80 | 5.6e-17 | 17.48 (0.719) | 20.64 (0.689) | 38.12 (0.687) | 0.459 (0.061) |
| | 1 PC | 41.96 | 4.7e-11 | 15.32 (0.714) | 22.57 (0.691) | 37.89 (0.686) | 0.404 (0.064) |
| | 5 PCs | 37.06 | 5.7e-10 | 14.90 (0.714) | 22.95 (0.691) | 37.85 (0.686) | 0.394 (0.066) |
| | 10 PCs | 37.58 | 4.4e-10 | 15.05 (0.714) | 22.82 (0.691) | 37.87 (0.686) | 0.397 (0.066) |
| | 20 PCs | 37.02 | 5.8e-10 | 15.05 (0.716) | 22.82 (0.691) | 37.87 (0.687) | 0.398 (0.066) |
| Weight | 0 PC | 13.92 | 9.6e-5 | 28.24 (2.32) | 97.23 (2.32) | 125.47 (2.11) | 0.225 (0.062) |
| | 1 PC | 13.80 | 1.0e-4 | 28.26 (2.32) | 97.21 (2.32) | 125.47 (2.11) | 0.225 (0.062) |
| | 5 PCs | 13.07 | 1.5e-4 | 27.90 (2.32) | 97.53 (2.32) | 125.43 (2.12) | 0.222 (0.062) |
| | 10 PCs | 13.45 | 1.2e-4 | 28.38 (2.33) | 97.11 (2.32) | 125.49 (2.11) | 0.226 (0.063) |
| | 20 PCs | 12.41 | 2.1e-4 | 27.55 (2.33) | 97.82 (2.32) | 125.38 (2.12) | 0.220 (0.063) |
| BMI | 0 PC | 10.44 | 6.2e-4 | 3.23 (0.302) | 13.09 (0.302) | 16.31 (0.304) | 0.198 (0.063) |
| | 1 PC | 9.34 | 1.1e-3 | 3.12 (0.302) | 13.18 (0.302) | 16.30 (0.304) | 0.191 (0.064) |
| | 5 PCs | 9.65 | 9.4e-4 | 3.18 (0.302) | 13.12 (0.303) | 16.31 (0.304) | 0.195 (0.064) |
| | 10 PCs | 10.21 | 7.0e-4 | 3.29 (0.303) | 13.04 (0.303) | 16.32 (0.304) | 0.201 (0.064) |
| | 20 PCs | 8.26 | 2.0e-3 | 3.00 (0.303) | 13.28 (0.303) | 16.28 (0.304) | 0.184 (0.065) |
| Head | 0 PC | 9.85 | 8.5e-4 | 0.722 (0.078) | 3.52 (0.079) | 4.24 (0.080) | 0.170 (0.057) |
| Circumference | 1 PC | 3.70 | 0.027 | 0.495 (0.078) | 3.73 (0.079) | 4.23 (0.080) | 0.117 (0.062) |
| | 5 PCs | 2.67 | 0.051 | 0.437 (0.078) | 3.79 (0.079) | 4.22 (0.079) | 0.103 (0.064) |
| | 10 PCs | 2.46 | 0.058 | 0.424 (0.078) | 3.80 (0.079) | 4.22 (0.080) | 0.100 (0.065) |
| | 20 PCs | 1.88 | 0.085 | 0.376 (0.078) | 3.84 (0.079) | 4.22 (0.079) | 0.089 (0.065) |
| Waist | 0 PC | 13.09 | 1.5e-4 | 9.2e-04 (8.6e-5) | 3.6e-3 (8.7e-5) | 4.5e-3 (8.7e-5) | 0.205 (0.061) |
| to | 1 PC | 3.54 | 0.030 | 5.6e-04 (8.6e-5) | 3.9e-3 (8.7e-5) | 4.5e-3 (8.7e-5) | 0.126 (0.068) |
| Hip | 5 PCs | 3.98 | 0.023 | 6.0e-04 (8.6e-5) | 3.9e-3 (8.7e-5) | 4.5e-3 (8.7e-5) | 0.133 (0.068) |
| Ratio | 10 PCs | 3.65 | 0.028 | 5.6e-04 (8.6e-5) | 3.9e-3 (8.7e-5) | 4.5e-3 (8.7e-5) | 0.125 (0.066) |
| | 20 PCs | 3.41 | 0.032 | 5.6e-04 (8.6e-5) | 3.9e-3 (8.7e-5) | 4.5e-3 (8.7e-5) | 0.126 (0.070) |

Table S8: Model parameter estimates for the anthropometric phenotypes and their standard error, depending on the number of PCs included in the model, likelihood ratio test statistics (LRT) to test significance of heritability and their $p$-values. $\widehat{\tau}$ is the estimated genetic variance, $\widehat{\sigma^2}$ the estimated residual variance, $\widehat{\sigma^2_P} = \widehat{\tau} + \widehat{\sigma^2}$ the estimated total variance, and $\widehat{h^2} = \widehat{\tau}/(\widehat{\tau} + \widehat{\sigma^2})$ estimated heritability.
